# The Changing Global Epidemiology of Early-Onset Colorectal Cancer: Incidence Trends, Modifiable Risk Factors, and Future Burden Projections

**DOI:** 10.3390/cancers18213453

**Published:** 2026-10-26

**Authors:** Kacper Wrzosek, Agata Koba, Pola Bakalczuk, Julia Oziębło, Karolina Michno, Weronika Pająk, Jakub Kleinrok, Agnieszka Korolczuk, Daria Trojanowska, Alicja Forma

**Affiliations:** 1 Chair and Department of Clinical Pathomorphology, Medical University of Lublin, 20-090 Lublin, Poland; kacperwrzosek2003@gmail.com (K.W.); agata.koba22@gmail.com (A.K.); bakalczukpola@gmail.com (P.B.); jozieblo001@gmail.com (J.O.); klejs.90@gmail.com (J.K.); 2 Student Scientific Society of Clinical Pathomorphology, Medical University of Lublin, 20-090 Lublin, Poland; 3 Student Scientific Society of Forensic Medicine, Medical University of Lublin, 20-090 Lublin, Poland; 4 Student Scientific Society of Correct, Clinical, and Imaging Anatomy, Medical University of Lublin, 20-090 Lublin, Poland; karolina.michno@stud.umed.lodz.pl; 5 University Clinical Hospital No. 2 of the Medical University of Lodz, 90-549 Lodz, Poland; 6 Faculty of Medicine, Lazarski University, 02-662 Warsaw, Poland; dariatrojanowska@icloud.com; 7 Department of General and Pediatric Ophthalmology, Medical University of Lublin, 20-059 Lublin, Poland; alicja.forma@umlub.edu.pl; 8 Department of Correct, Clinical and Imaging Anatomy, Medical University of Lublin, ul. Jaczewskiego 4, 20-090 Lublin, Poland

**Keywords:** early-onset colorectal cancer, global epidemiology, incidence trends, modifiable risk factors, lifestyle factors, future disease burden, cancer prevention

## Abstract

Early-onset colorectal cancer (EOCRC), defined as colorectal cancer diagnosed before age 50, has become an increasing global public health concern. Incidence rates have risen among younger adults in many countries, despite stable or declining rates in older populations. This review examines global EOCRC epidemiology, potentially modifiable risk factors, and projected future disease burden. Evidence from international cancer databases, population-based registries, and observational studies is synthesised, considering differences by age, sex, geographic region, and tumour location. Key risk factors include obesity, metabolic dysfunction, dietary patterns, physical inactivity, alcohol consumption, tobacco use, antibiotic exposure, and gut microbiome alterations. The review also evaluates temporal trends and projections of incidence, prevalence, and mortality. Although increasing incidence may reflect changing lifestyles, metabolic health, environmental exposures, and other early-life risk factors, their relative contributions remain incompletely understood. Improved understanding of EOCRC epidemiology and future burden may help identify high-risk populations, guide targeted prevention strategies, inform public health policy, and support discussions on earlier colorectal cancer detection and screening.

## Simple Summary

Colorectal cancer diagnosed before the age of 50 is becoming an increasingly serious public health problem in many parts of the world. Although colorectal cancer is traditionally associated with older adults, its incidence among younger people is rising in several populations, highlighting the need for a better understanding of the factors driving this change. This review analyses global trends in early-onset colorectal cancer, including differences across geographic regions, age groups, and genders. Potentially modifiable risk factors, such as obesity, unhealthy dietary habits, physical inactivity, alcohol consumption, smoking, and changes in the gut microbiome, are also examined. In addition, projections regarding the future disease burden and their implications for prevention and healthcare planning are discussed. By compiling current scientific evidence, this review aims to identify gaps in knowledge, support the development of targeted prevention strategies, and guide future research into the causes of early-onset colorectal cancer.

## 1. Introduction

Colorectal cancer (CRC) is the third most commonly diagnosed malignancy worldwide and the second leading cause of cancer death. In 2022, it accounted for an estimated 1,926,425 new cases and 904,019 deaths [1]. Within this burden, early-onset colorectal cancer (EOCRC), conventionally defined as CRC diagnosed before the age of 50 years, has emerged over the past three decades as a distinct epidemiological problem. The 50-year threshold is pragmatic rather than biological, and the literature applies it inconsistently. One of the largest prospective analyses defines early onset as diagnosis before 55 years [2], and studies of metabolic comorbidity have used age 60 as the boundary of the “younger” stratum [3]. This heterogeneity complicates direct comparison between studies.

EOCRC differs clinically and epidemiologically from average-onset disease. Tumours arising before 50 years are predominantly left-sided [4] and present at a more advanced stage, both in US registry data [5] and in an international meta-analysis in which 60% of early-onset cases, compared with 49% of late-onset cases, were diagnosed at stage III or IV [6]. The molecular profile differs in degree rather than kind, with modest shifts in the frequency of common driver mutations and microsatellite instability, whose direction is not consistent across series [4,7]. Diagnosis before 50 also carries consequences that incidence counts do not capture, including employment disruption and caregiving burden, unmet fertility and sexual health needs, and persistent treatment-related morbidity among survivors [5].

The epidemiological signal that defines EOCRC is not its absolute incidence, which remains far below that of older adults, but its divergence from the overall CRC trend. In the United States, incidence rose in adults aged 20 to 49 years while falling in those aged 65 and over between 2013 and 2022 [5]. Mortality has moved in the same direction, with CRC advancing from the fifth to the first leading cause of cancer death among US adults under 50 years between the early 1990s and 2023, a rank change that reflects both rising CRC mortality and a 44% fall in overall cancer mortality in this age group [8]. The same divergence appears internationally: early-onset incidence rose in 27 of 50 countries and territories and declined in none. At the same time, rates in adults aged 50 to 74 years were stable or fell in more than half of those populations. However, the country with the highest rate is not among those rising fastest [9]. Mortality among adults aged 25 to 49 years has risen in the United Kingdom while falling across the EU-27 [10].

Age–period–cohort analysis locates the increase in birth cohorts rather than in calendar periods, with risk rising in successive generations born from the mid-1950s onwards and the steepest increases in the youngest strata. However, a period component is also present [11]. Pathogenic germline variants are reported in 12% to 22% of EOCRC, depending on the age threshold and panel size [12,13,14], and the authors interpret the cohort pattern as consistent with exposure beginning in childhood or adolescence [11].

Part of the observed increase may be an artefact of detection or classification. Earlier screening initiation in the United States and the reclassification of neuroendocrine neoplasms both inflate recent registry counts [15,16,17], but neither accounts for the rise at ages 20 to 39 years, which lies outside any screening programme and was evident well before the guideline change [11].

For the modifiable exposures implicated by the cohort pattern, available contribution estimates remain single-factor or model-derived [18,19]. One risk-factor analysis attributes 88.7% of early-onset but 97.6% of late-onset disability-adjusted life years to eleven measured factors, leaving a larger residual in the younger group. However, those estimates apply the same relative risks at every age [20]. The projection literature is similarly fragmented, with published forecasts of total CRC burden ranging from a 22.5% increase by 2050 to 70% by 2045, and with one model projecting a peak and subsequent decline [11,20,21,22,23].

This review therefore pursues three objectives. First, we describe the changing global epidemiology of EOCRC, separating incidence level from incidence trend and true change from ascertainment artefact. Second, we assemble the published attributable fractions for established modifiable exposures, show why they cannot be combined into a single estimate of how much of the observed birth-cohort effect they account for, and state what such an estimate would require. Third, we compare the available projection models and identify the methodological choices that explain why their estimates disagree.

Accordingly, the review addresses three questions. How are global incidence patterns of EOCRC changing? Which modifiable risk factors may contribute to these trends? What can be expected regarding the future burden of disease?

## 2. Materials and Methods

This article is a narrative review of published evidence. It was not registered, no protocol was published in advance, and it does not meet the criteria for a systematic review; therefore, the PRISMA statement does not apply, and no flow diagram of study selection is presented. No formal risk-of-bias instrument was applied to individual studies, and no meta-analysis was undertaken. Because only published aggregate data are used, neither ethical approval nor informed consent was required.

We identified peer-reviewed articles by searching PubMed/MEDLINE, Scopus and Web of Science, together with the websites of the journals in which this literature appears, and we used the reference lists of the retrieved articles to find additional sources. No lower date limit was applied, and the last search was run in October 2026. The searches combined terms for early-onset or young-onset colorectal cancer with terms for incidence, temporal trends, birth cohort and age–period–cohort analysis, modifiable risk factors, and projections of future burden. Only publications in English were considered. Preference was given to analyses reporting age-specific results for adults below 50 years of age, to population-based rather than hospital-based series, and to analyses covering more than one country or registry. When sources disagreed, we consulted the primary analysis rather than a secondary source that cited it.

We did not compute rates, trends, or effect estimates for this review, and we did not extract new data from cancer surveillance databases. Every value reported here is taken from a published analysis, is named as that analysis names it, whether an annual per cent change, an average annual per cent change or an estimated annual per cent change, and is given with the age range and the observation period to which it refers. An average annual per cent change is defined only over the interval for which it was computed [24], so values with different names, or computed over different periods, are not treated as measures of the same quantity, and none is converted into another, pooled, or averaged. An incidence rate ratio between two age strata is reported as a difference between age groups and not as a change over time. Incidence rates are compared numerically only within a source, and rates from different sources are set beside one another only to indicate the direction and the spread of the evidence.

Early-onset colorectal cancer is taken throughout as colorectal cancer diagnosed before 50 years of age. The primary sources use different age bounds, including 15 to 49, 20 to 49, and 25 to 49 years, so each source states the age range used when reporting results and rates derived from different age ranges are not pooled.

Where a reported increase in incidence might reflect a change in detection or registration rather than in the occurrence of disease, the published evidence is examined for whether the increase persists after exclusion of tumour types whose registration rules changed, whether it is present in age groups that lie outside any screening programme, whether it extends to advanced-stage disease, and how mortality moved over the same period.

## 3. Global Epidemiology of EOCRC

### 3.1. Global Incidence

Zhang et al. reported a global age-standardised incidence of EOCRC of 5.37 per 100,000 in 2021, corresponding to an average annual percentage change (AAPC) of 0.99% since 1990 [25]. Other Global Burden of Disease (GBD)-based analyses report different values, and Section 7.2 discusses the reasons for these differences.

Global patterns of EOCRC also vary by gender. In 2021, the incidence rate was higher among men than among women (6.33 vs. 4.37 per 100,000), and the long-term growth rate was also higher among men (AAPC 1.31% vs. 0.57%) [25].

However, this pattern is not uniform across all populations. In the GBD 2019 analysis, in countries with a low socio-demographic index (SDI), incidence rates were similar for both sexes, whilst the growth rate was higher among women than men (APC 1.54% vs. 1.12%) [26]. This suggests that gender differences in EOCRC epidemiology may depend on population and socio-demographic context.

Differences in EOCRC epidemiology also relate to the tumour’s anatomical location. In an international registry-based analysis by Siegel et al., the incidence of colon cancer was higher than that of rectal cancer in most of the populations studied; however, this pattern was not uniform: in Korea, India and China, the incidence of rectal cancer was slightly higher, whilst in Slovenia the figures for both sites were similar [27].

Rectal localisation also shows up in temporal trends. In a systematic review comprising 40 studies from 12 countries, 9 out of 14 studies analysing tumour site showed an increase in the incidence of rectal cancer in people under 50 years of age, with an APC of 4.03 per cent; this trend was particularly evident in data from North America and Australia [28].

Tumour location within the colon also varies. In an analysis of 21 population-based registries from Australia, Canada, Denmark, Norway, New Zealand, Ireland and the United Kingdom, the increase in colon cancer incidence in people under 50 was primarily associated with a rise in distal, left-sided colon tumours [29]. However, this pattern was not observed in all populations. In a German analysis covering the period 1999–2018, the sharpest increase was seen in cancer of the proximal colon, whilst the incidence of cancer of the distal colon remained essentially stable [30]. Taken together, these data indicate that the anatomical pattern of EOCRC is heterogeneous: rectal and distal colon cancer increase in many populations, but the direction of change by location is not universal.

### 3.2. Temporal and Age-Specific Trends

An earlier international analysis of registry data from 36 countries demonstrated increasing EOCRC incidence in 19 populations. In most high-income countries with long-term data, the increase began around the mid-1990s. A more recent analysis by Sung et al., covering 50 countries and territories, confirmed and extended this pattern. During the most recent decade analysed, EOCRC incidence increased in 27 of 50 populations, while remaining stable in the other 23. Importantly, the increase was no longer confined to high-income Western countries but was also observed in several populations in Eastern Europe, Asia, and Latin America and the Caribbean [9,27].

In 14 of the 27 populations with increasing EOCRC incidence, incidence among individuals aged 50–74 years remained stable or decreased. In another six populations, incidence increased in both age groups but rose more rapidly among younger adults. Thus, in 20 of the 27 populations, incidence increased either in younger adults or more rapidly than in older adults, indicating marked age-related heterogeneity in incidence trends [9].

#### 3.2.1. Birth-Cohort Perspective

Birth-cohort analyses suggest that, in some populations, EOCRC increases may be more strongly associated with cohort effects than with period effects. In Chen and Yang’s analysis, which included registry data from 35 countries, cohort effects clearly predominated in Shanghai, the United Kingdom, Australia, and New Zealand. In Canada, the United States, and Osaka, both cohort and period effects were observed, although quantitative analysis also indicated a predominance of cohort effects. The timing of these changes varied geographically: in the United States, the turning point was observed among cohorts born in the 1940s–1950s; in Canada and Australia, among those born in the 1950s–1960s; in New Zealand and Osaka, among cohorts born in the 1960s; and in Shanghai, among those born in the 1970s–1980s. Thus, across the populations analysed, turning points in cohort effects generally occurred earlier in Western countries than in Shanghai and Osaka [31].

Birth-cohort effects have also been observed in Latin American populations, which remain substantially underrepresented in international analyses. An age–period–cohort analysis using population-based cancer registry data from Cali (Colombia), Costa Rica, Goiânia (Brazil), and Quito (Ecuador) identified cohort effects in CRC incidence in most of the populations analysed. For CRC overall, increasing relative risks across successive birth cohorts were observed from approximately the 1939 birth cohort in Cali and Costa Rica and from approximately the 1943 birth cohort in Goiânia. In contrast, in Quito a cohort effect was identified only among men [32]. However, the analysis included individuals aged 20–79 years and was not restricted to EOCRC; therefore, these findings should be regarded as regional complementary evidence of birth-cohort effects rather than as direct estimates of EOCRC trends. The pattern also varied by anatomical subsite. For rectal cancer, increasing birth-cohort-related risks in more recent cohorts were observed, among others, in both sexes in Cali, among men in Costa Rica, and among women in Goiânia [32].

Further evidence comes from age–period–cohort analyses based on data from 21 population-based cancer registries in seven high-income countries. These analyses demonstrated strong, nonlinear birth-cohort effects that were more pronounced for rectal than for colon cancer. Combined with increasing incidence among individuals aged < 50 years and simultaneous declines among those aged 50–74 years, this pattern indicates that incidence changes have not affected successive generations uniformly but have been particularly pronounced in more recent birth cohorts [29].

An analysis of Australia, Canada, England, and the United States illustrates the magnitude of differences between successive birth cohorts. The risk began to increase among successive generations born around 1955 in the United States and around 1960 in Australia, Canada, and England. Compared with men born in 1960, those born in 1995 had an approximately fourfold higher risk in the United States and up to an eightfold higher risk in England, with even higher estimates observed among women [11].

Authors of analyses including Australia, Canada, England, and the United States suggest this pattern may reflect exposures beginning as early as childhood or adolescence, with effects that persist or accumulate later in life [11]. However, this interpretation is an etiological hypothesis based on the cohort effect rather than direct evidence of the contribution of specific exposures.

#### 3.2.2. Age-Specific Trends

The increase in EOCRC incidence is not uniform across the entire age range below 50 years but shows a clear age-related gradient. The largest increases occur among the youngest adults, and the magnitude gradually declines as age approaches 50 years. In an analysis including Australia, Canada, England, and the United States, the steepest incidence increases were among individuals aged 20–24 and 25–29 years, clearly exceeding those observed in the 40–44 and 45–49-year age groups [11].

These differences also appear when examining the rate of change over the most recent decade for which data were available. Between 2008 and 2017, the mean estimated annual percentage change in incidence was 7.0% among individuals aged 20–39 years, compared with 3.7% in the overall population aged 20–49 years. All four countries analysed showed an increase among individuals younger than 40 years, with estimated annual percentage change ranging from 5.0% in the United States to 9.1% in England. The mean rate of increase among individuals aged 20–39 years was also greater among women than among men (8.4% vs. 5.6% per year) [11].

**Figure 1:**
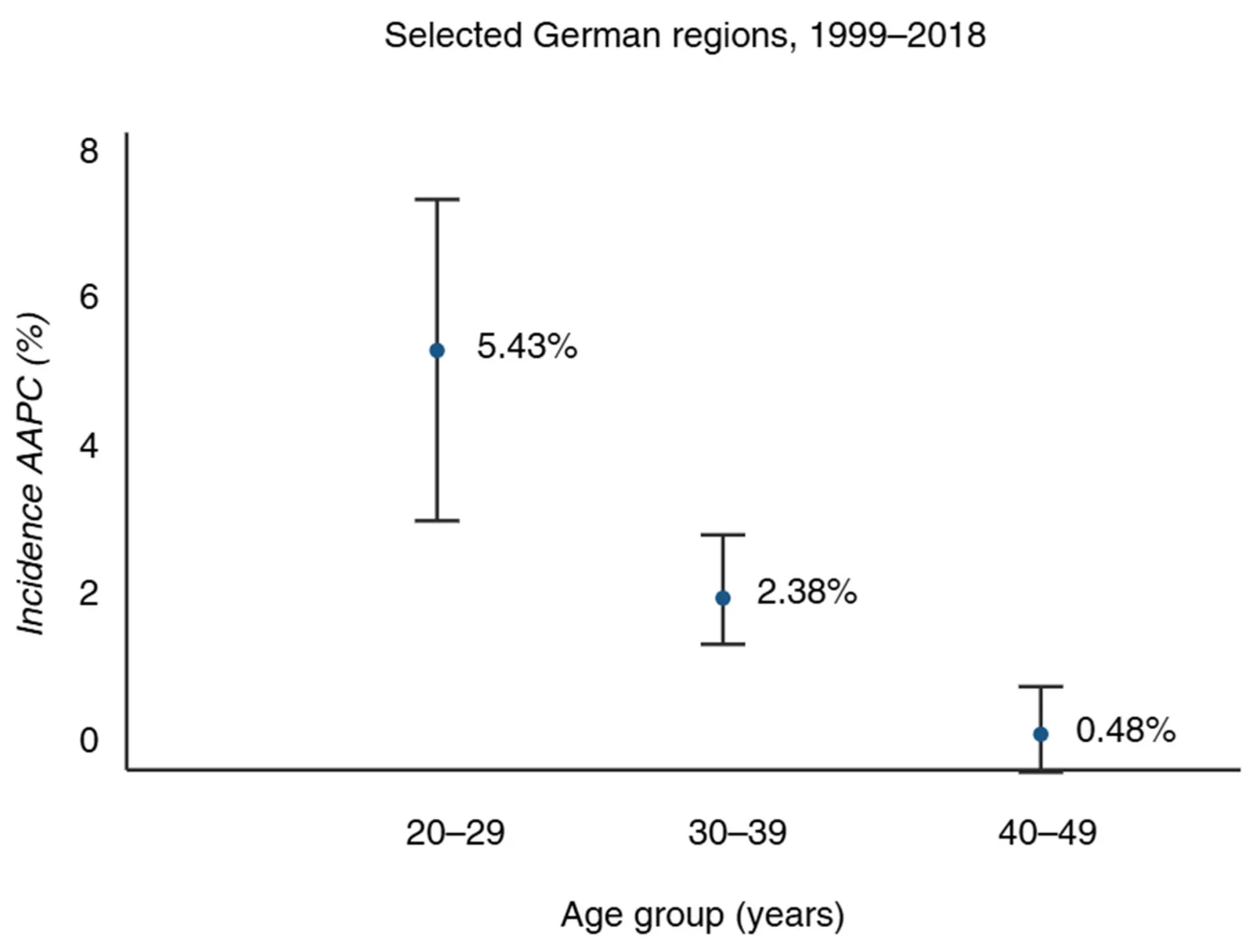
Age-specific trends in early-onset colorectal cancer incidence in selected German regions, 1999–2018. Points represent the average annual percentage change (AAPC) in incidence for both sexes combined among individuals aged 20–29, 30–39, and 40–49 years; error bars indicate 95% confidence intervals—data from Tanaka et al. [30].

Germany showed a similar age gradient between 1999 and 2018. The largest increase occurred among individuals aged 20–29 years, with AAPCs of 4.44% in men and 6.40% in women. Among those aged 30–39 years, the increase was smaller but remained statistically significant and exceeded 2% per year in both sexes, whereas incidence rates among individuals aged 40–49 years remained essentially stable [30]. These findings independently confirm that, in some populations, the increase in EOCRC is concentrated primarily among the youngest adults and gradually attenuates with increasing age (Figure 1).

However, the age gradient varies across populations. Guam Cancer Registry data from 1998 to 2020 showed that the largest relative differences in incidence rates between successive age strata occurred before age 50. The incidence rate among individuals aged 40–44 years was 2.01 times higher than among those aged 35–39 years, while the incidence rate among individuals aged 45–49 years was 1.99 times higher than among those aged 40–44 years (incidence rate ratio (IRR) 2.01; 95% CI, 1.14–3.53 and 1.99; 95% CI, 1.34–2.97, respectively) [33]. These findings should not be interpreted as rates of change in incidence over time, but rather as differences in incidence between successive age groups.

We also observed heterogeneity across ethnic groups in Guam. Among CHamoru individuals, the largest relative difference between successive age groups occurred between ages 40–44 and 45–49 years (IRR 2.11), whereas among Filipino individuals, it occurred only between ages 55–59 and 60–64 years (IRR 1.76) [33]. This indicates that the age gradient may not follow the same pattern across populations and that aggregated data may partially obscure differences between population subgroups.

### 3.3. Geographic Heterogeneity

**Figure 2:**
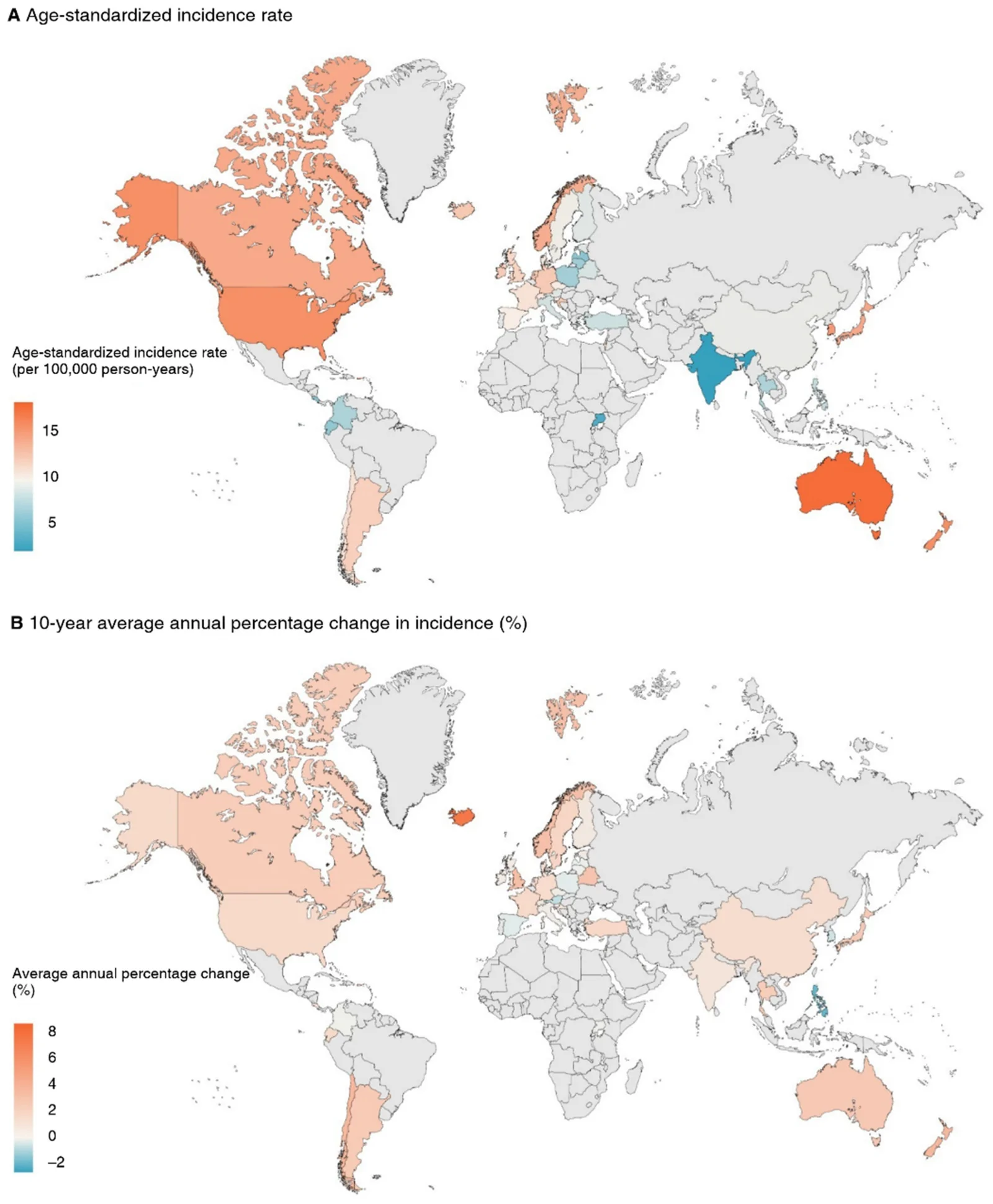
Geographic variation in early-onset colorectal cancer incidence and recent incidence trends among adults aged 25–49 years. (**A**) Age-standardised incidence rates (ASRs) per 100,000 person-years for the most recent 5 years of available data (2013–2017, except Japan [2011–2015], Spain [2012–2016], and Costa Rica [2012–2016]). (**B**) Most recent 10-year average annual percentage change (AAPC) in age-standardised incidence rates (2008–2017, except Japan [2006–2015], Spain [2005–2016], and Costa Rica [2005–2016]). Data come from national or subnational population-based cancer registries; therefore, geographic coverage varies by country. Grey areas indicate countries or territories not included in the analysis. Malta is included in the source data but is not displayed because of map resolution—data adapted from Sung et al. [9].

EOCRC incidence shows substantial geographic variation in both current incidence levels and the direction and magnitude of temporal trends (Figure 2). In the international analysis by Sung et al., which included 135 cancer registries from 50 countries and territories, the age-standardized incidence rate (ASR) among individuals aged 25–49 years was highest in Australia (16.5 per 100,000 person-years), Puerto Rico (15.2), New Zealand and the United States (14.8 each), and South Korea (14.3). In contrast, India (3.5) and Uganda (4.4) had the lowest rates [9]. An earlier international analysis also showed substantial differences between populations, with incidence rates among individuals aged 20–49 years during 2008–2012 ranging from 3.5 per 100,000 in Chennai, India, to 12.9 per 100,000 in Korea [27]. Differences in age ranges and observation periods between studies limit direct comparisons of incidence rates; nevertheless, both datasets demonstrate substantial geographic heterogeneity in the burden of EOCRC [9,27].

Similar variation is observed across world regions. According to GBD 2021 estimates [25], East Asia had the highest incidence rate among the 21 regions studied (11.86 per 100,000), and Western sub-Saharan Africa had the lowest (0.82 per 100,000). However, the areas with the highest incidence were not necessarily the fastest-growing areas. Central Latin America had the largest increase over the long period between 1990 and 2021 (AAPC 3.48%), and East Asia had the highest incidence in 2021, but its AAPC was 2.67%. Incidence did not change significantly compared with Central Asia (AAPC, −0.28%; 95% CI, −0.83 to 0.27) or Eastern sub-Saharan Africa (AAPC, −0.02%; 95% CI, −0.15 to 0.11) [25]. These findings suggest that the regions bearing the highest current burden of EOCRC are not necessarily those with the fastest epidemiological transition.

The increase in EOCRC was not confined to high-income Western countries. Sung et al. reported rising incidence across multiple Latin American and Caribbean populations, including Argentina, Chile, Costa Rica, Ecuador, Martinique, and Puerto Rico [9]. Chile showed particularly rapid increases, with an AAPC of 3.96% (95% CI, 1.26–6.74), one of the highest reported across the analysed populations [9]. For population-based data from Uruguay, the trend may differ in younger age groups within individual Latin American populations. The nationwide cancer registry (2002–2017) showed a significant increase in CRC incidence in both men (APC 3.10%; 95% CI, 1.21–5.03) and women (APC 2.06%; 95% CI, 0.49–3.66) in the age group 40–49 years, without significant changes in the age group 20–39 years [34].

Incidence also varies by sociodemographic development level. In 2021, high-SDI regions had the highest incidence rate (10.15 per 100,000), followed by high-middle-SDI regions (9.87 per 100,000), whereas low-SDI regions had 1.30 per 100,000 [25]. However, the rate of change did not match the current incidence level. Over 1990–2021, high-middle-SDI (AAPC 1.96%) and middle-SDI (AAPC 1.79%) regions saw the fastest increases, whereas the long-term trend in low-SDI regions remained essentially stable (AAPC −0.02%; 95% CI, −0.16 to 0.12) [25].

However, an analysis restricted to a shorter, more recent period showed a different pattern in low-SDI countries. Between 2010 and 2019, the EOCRC incidence rate in low-SDI countries increased by an average of 1.33% per year, compared with 0.95% globally [26]. Despite the more rapid increase, the absolute incidence rate remained substantially lower than the global average: in 2019, the corresponding rates were 1.43 and 5.74 per 100,000, respectively [26]. This discrepancy may reflect differences in the periods analysed and suggest a more rapid increase in recent years; however, methodological differences between the studies preclude direct inference about a change in the trend’s direction.

Cancer registry data from sub-Saharan Africa show that incidence trends may vary across populations. In an analysis including 12 registries from 11 countries, CRC incidence increased in all populations with continuous time-series data, with the highest AAPCs observed in Brazzaville and Ibadan (6.1% per year in both populations) [35]. However, the analysis was not restricted to EOCRC, and the age-specific comparison was based on individuals aged < 55 vs. ≥55 years. In most registries, the increase was more pronounced in the older age group, while changes among individuals aged < 55 years did not reach statistical significance in five populations [35]. These findings indicate an increasing burden of CRC in some sub-Saharan African populations but do not allow a definitive conclusion that EOCRC is increasing in parallel.

EOCRC heterogeneity extends beyond population differences to include changes in temporal trajectories. In Sung et al.’s analysis, previously increasing incidence trends in Croatia and Germany stabilised during the most recent five-year period [9]. At the same time, compared with earlier analyses, previously stable trends in Croatia, France, and Ireland shifted toward increasing incidence, whereas previous declines in Austria, Italy, and Lithuania stabilised [9]. EOCRC trend estimates may differ across studies. For example, Sung et al. reported stable incidence in Croatia over the most recent five-year period, whereas a longer analysis showed an increasing trend [9].

Taken together, the available evidence indicates that the global epidemiology of EOCRC does not follow a single universal pattern. High current incidence does not necessarily coincide with the fastest increase, and trend directions may differ across regions and countries and may also change over time within the same population [9,25,26,34]. Geographic heterogeneity in EOCRC therefore encompasses three dimensions: current incidence, rate of change, and the direction and evolution of trends over time.

### 3.4. Is the Rise Real? Ascertainment and Classification Artefacts

Interpretation of international trends in EOCRC is limited by variable availability of registry data. Sung et al. found that 23 of the 50 populations were represented solely by subnational registries and that countries in Africa and Asia were particularly underrepresented. Moreover, limited data on tumour anatomical subsite limited our ability to assess whether the observed trends were consistent across CRC subtypes. These limitations should be considered in light of the magnitude and geographic extent of the increase in EOCRC [9].

Changes may also influence observed trends in cancer detection and screening uptake. However, the particularly rapid incidence increase among individuals aged 20–29, 30–34, and 35–39 years, with weaker increases among those aged 40–44 and 45–49 years, is difficult to explain by screening alone [11]. The incidence increase observed before age 30 indicates that the phenomenon is not confined to age groups closest to the eligibility threshold for population-based screening.

Comparing age-specific patterns in Guam and the continental United States suggests that screening may influence observed incidence rates. In Guam, the largest relative differences in incidence rates between successive age groups occurred before age 50, whereas in the United States the largest difference was observed between the 45–49 and 50–54-year age groups (IRR 1.90; 95% CI, 1.86–1.95) [33]. The authors suggested that the pattern observed in the United States may be partly related to cancers detected at the initiation of screening. However, the study did not quantify the magnitude of this effect [33].

More recent data from German cancer registries further indicate that earlier detection may partly influence the observed EOCRC trends. Between 2003 and 2023, after excluding appendiceal tumours, increasing incidence was concentrated among individuals aged 20–29 and 30–39 years, whereas rates among those aged 40–49 years remained essentially stable [36]. The increase largely involved tumours with more favourable prognostic features, including T1 tumours and low-grade lesions; the incidence of neuroendocrine neoplasms (NENs) also increased. Given nearly stable mortality, the authors considered this pattern consistent with a partial contribution from earlier detection [36]. At the same time, T4 tumour incidence also increased among men and women aged 30–39 years, suggesting that increased detection does not fully explain the observed trend [36].

Independent of detection changes, estimates of EOCRC trends may also be influenced by changes in how specific tumour types are classified and registered. In an analysis of US data, excluding appendiceal cancer reduced the AAPC for CRC among individuals aged 20–49 years from 2.2% to 1.7% and that for colon cancer from 2.1% to 1.3%, although the increase remained after these tumours were excluded; similar findings were reported for Canada and New Zealand [27]. In contrast, an earlier German analysis showed that after excluding appendiceal tumours, the previously observed increase in colon cancer incidence was no longer statistically significant [30]. The authors also suggested that changes in the coding of appendiceal carcinoid tumours may have contributed to this effect [30]. However, a more recent German analysis covering 2003–2023 showed a persistent moderate increase in overall EOCRC after excluding appendiceal tumours [36]. Thus, the two analyses are not directly contradictory; they suggest that appendiceal tumours may substantially affect the estimated trend magnitude without accounting for the full observed increase in EOCRC.

The mechanism by which classification changes may influence observed trends is particularly evident in data from French cancer registries, where adenocarcinomas (ADCs) and NENs were analysed separately. Among individuals aged 15–39 years, CRC incidence, including all histological types, increased, whereas ADCs alone showed no significant increase. NENs accounted for 29.7% of all CRCs in the 15–39-year age group, compared with 5.7% among those aged 40–49 years, and their incidence increased sharply between 2004 and 2013 [17]. This period included changes in NEN classification and registration criteria that expanded registration to some tumours previously not classified as invasive malignancies. This phenomenon was particularly evident in the youngest age group because NENs made up a large proportion of registered CRCs [17].

However, classification changes do not eliminate all signals indicating an increasing disease burden. In the French analysis, the incidence of nonmetastatic ADCs M0 remained essentially stable, whereas a modest upward trend was observed for metastatic ADCs M1 in younger age groups [17]. Similarly, the increasing incidence persisted after excluding appendiceal tumours in analyses from the United States, Canada, and New Zealand [27], as well as in more recent German data [36].

Taken together, the available evidence suggests that differences in registry completeness, the intensity of diagnostic investigations and screening, and changes in tumour classification and registration may partly explain the observed increase in EOCRC. These factors may primarily affect the magnitude of the observed increase without necessarily accounting for its occurrence. The persistence of increasing incidence after excluding tumours particularly susceptible to classification changes, its presence in the youngest age groups, and the increase in T4 tumours or metastatic adenocarcinomas observed in some analyses indicate that the phenomenon cannot be attributed entirely to epidemiological artefacts [11,17,27,36].

## 4. Changing Distribution of EOCRC

Section 3 established where and how fast early-onset incidence is rising. This section asks what those tumours are. It compares the anatomical, pathological, hereditary, and molecular composition of early-onset disease with that of later-onset disease, and asks whether that composition is changing over time. Trends in subsite-specific incidence among younger adults belong to Section 3.1 and are not repeated here.

### 4.1. Anatomical Subsite

Early-onset tumours are more often distal. In the Surveillance, Epidemiology, and End Results database for 2004 to 2015, which covered 240,772 patients with colorectal cancer, distal tumours accounted for 70.6% of cases diagnosed at 20 to 49 years, compared with 57.6% diagnosed at 50 to 79 years [37]. In the same analysis, a rectal or caecal primary was independently associated with higher cause-specific mortality, so subsite carries prognostic as well as descriptive weight [37].

The shift towards the rectum is not confined to young adults. In United States registry data for all ages combined, rectal cancer accounted for 32% of colorectal cancers in 2022 against 27% in 2004, and among adults aged 65 years and over rectal cancer has become 21% more common than distal colon cancer, reversing the historical pattern [5]. The distal predominance of early-onset disease is therefore a difference in degree against a background that is moving in the same direction at all ages. The pattern also holds after disease progression. Among 23,278 patients with metastatic colorectal cancer recorded in the same registry between 2010 and 2020, left colon tumours accounted for 49.63% of early-onset cases against 38.23% of average-onset cases [38].

### 4.2. Stage at Diagnosis

Early-onset disease is diagnosed later. In United States registry data, three in four patients younger than 50 years present with advanced disease (regional or distant stage), and 27% present with distant metastases, compared with 21% to 23% among older patients [5]. A meta-analysis of 26 studies comprising 1,062,037 patients found 60% of early-onset cases against 49% of late-onset cases diagnosed at stage III or IV, with a risk ratio of 1.26 and a 95% confidence interval of 1.19 to 1.35 [6]. The Surveillance, Epidemiology, and End Results analysis cited above reported 61.3% against 48.6% for advanced stage over the same age contrast [37], and a larger meta-analysis of 50 studies and 6,148,851 patients reported 22.6% of early-onset cases metastatic at diagnosis and a higher risk of stage IV disease [39]. The three estimates are not independent because the registry analyses feed the meta-analyses, and the definitions differ: a threshold set at regional or distant stage is not the same as one set at stage III or IV. Heterogeneity is also high, with an I-squared of 87% in the first meta-analysis and 97% in the second, so the pooled values indicate direction rather than magnitude [6,39].

The stage distribution is itself shifting. In an analysis restricted to adenocarcinoma in the Surveillance, Epidemiology, and End Results database, comparing 2000 to 2002 with 2014 to 2016, the proportion of rectal cancers presenting at distant stage rose from 18% to 31% among those aged 20 to 29 years and from 20% to 29% among those aged 30 to 39 years [40]. Over the same period, incidence of distant-stage rectal adenocarcinoma rose by 133% in the 20- to 29-year group and by 97% in the 30- to 39-year group, and distant-stage colon adenocarcinoma rose by 49% among those aged 30–39 years [40]. Because the analysis excluded neuroendocrine neoplasms, these shifts are not attributable to the reclassification discussed in Section 3.4.

Later stage has not translated into worse cancer-specific survival. The meta-analysis of 26 studies found better overall survival in early-onset disease (HR 0.89; 95% CI 0.81 to 0.99), but no difference in cancer-specific survival (HR 0.94; 95% CI 0.83 to 1.06) or disease-free survival (HR 1.05; 95% CI 0.94 to 1.16) [6]. Registry data give the same picture, with five-year disease-specific survival of 66.4% at 20 to 49 years against 68.2% at 50 to 79 years, a difference the authors report as not significant [37], and five-year relative survival for distant-stage disease of 23% below 50 years against 17% at 50 to 64 years and 10% at 65 years and over [5]. The advantage persists in metastatic disease, where median overall survival was 30 months in early-onset cases vs. 18 months in average-onset cases, with cause-specific survival showing the same pattern [38]. In that analysis, early-onset patients were also more likely to receive cancer-directed chemotherapy and radiotherapy, so the comparison reflects treatment intensity as well as host factors [38].

The favourable comparison does not extend to the youngest patients. Survival was lowest in the third decade of life at 59.0% [37], and a systematic review of 21 studies and 332,451 patients found that, when comparing several age groups, the youngest patients often had worse survival, particularly in stage II and stage III disease [41]. Heterogeneity prevented that review from pooling its results, so the finding is a consistent direction across studies rather than a quantified effect [41].

### 4.3. Hereditary vs. Sporadic Disease

The reported prevalence of pathogenic germline variants in early-onset disease ranges from about 1 in 8 to more than 1 in 5, and this range tracks the age threshold and the size of the gene panel. Among 450 unselected patients diagnosed before age 50 and tested on a 25-gene panel, 16% carried at least one pathogenic variant [12]. Among 3980 patients diagnosed between 15 and 49 years and tested on a 14-gene panel, 12.2% carried one, with values from 9.5% in Asian to 14.0% in Hispanic patients [13]. Among 268 French patients diagnosed before age 41 and tested with a 17-gene panel, 21.6% carried one [14].

Phenotype concentrates the yield. In the French series, pathogenic variants were found in 60.1% of mismatch repair-deficient cases and 48% of polyposis-associated cases, but only 2.3% of mismatch repair-proficient cases without polyposis [14]. In the unselected American series, 83.3% of patients with mismatch repair-deficient tumours carried a variant, compared with 8% of those with mismatch repair-proficient tumours, and one third of all carriers would not have met the established testing criteria for the gene in which their variant was found [12]. A clinicogenomic series with matched normal sequencing reported pathogenic germline variants in 6.9% of early-onset cases, compared with 5.0% of average-onset cases, a lower figure that reflects a cohort in which 80% of patients had stage 4 disease and in which matched normal material was available for fewer than three in five [4].

Two limits should be kept in view. These studies report prevalence at the time of testing, and none were designed to measure whether that prevalence has changed across birth cohorts, so they constrain rather than explain the trends described in Section 3. Taken at face value, they also place most early-onset disease outside the recognised high-penetrance syndromes.

### 4.4. Molecular Characteristics Relevant to Epidemiology

A meta-analysis of 149 studies compared tumour markers in early-onset and late-onset disease. Mutations in *KRAS*, *BRAF*, *APC* and *NRAS* were less common in early-onset tumours, with odds ratios of 0.91, 0.63, 0.70 and 0.88 and 95% confidence intervals of 0.85 to 0.98, 0.51 to 0.78, 0.58 to 0.84 and 0.78 to 1.00 respectively, while mutations in *PTEN* and TP53 were more common, at 1.68 and 1.34 with intervals of 1.04 to 2.73 and 1.24 to 1.45 [7]. The CpG island methylator phenotype was markedly less common (0.24; 0.10 to 0.57), and adverse histological features were more common, with odds ratios of 1.20 for high tumour grade, 1.22 for mucinous, and 2.32 for signet ring histology [7]. Restricting the analysis to studies that excluded Lynch syndrome reduced the associations for *BRAF* and *APC* to 0.77 and 0.81, respectively, and produced an inverse association for *PIK3CA* at 0.88 [7].

The direction of microsatellite instability is inconsistent across sources. The meta-analysis reported a positive association, with an odds ratio of 1.31 and an interval of 1.11 to 1.56 [7]. A clinicogenomic series of 11,006 patients, 2379 with early-onset disease, reported high microsatellite instability or deficient mismatch repair in 4.2% of early-onset tumours vs. 6.8% of average-onset tumours [4]. The two are not directly comparable because the meta-analysis includes series that did not exclude Lynch syndrome, and the clinicogenomic series is heavily weighted toward stage 4 disease. Still, the discrepancy is large enough that a single figure for microsatellite instability in early-onset disease should not be quoted without its source.

A third source addresses the question within rectal cancer and shows why a single prevalence figure would in any case be the wrong summary. In the National Cancer Database for 2004 to 2019, which covers 48,407 rectal adenocarcinomas, 8383 were diagnosed before age 50; among these, 663 had high microsatellite instability, compared with 2377 in late-onset cases, and the study did not test whether their proportion differs between the two age groups [42]. It does show that the clinical meaning of high microsatellite instability differs by age. Among early-onset cases, these tumours presented less often at stage IV (17.7% vs. 22.1%) and with liver metastasis less often (9.1% vs. 13.5%). In contrast, among late-onset cases, stage and metastatic pattern did not differ by microsatellite status [42]. In both age groups, they were more often pathological T3 or T4, mucinous and poorly differentiated, and overall survival did not differ by microsatellite status in either group [42].

Two further observations bear on interpretation. Mutation frequencies in early-onset non-hypermutated tumours differ by race, ethnicity and sex, including for *APC*, *PIK3CA*, *FBXW7*, *RNF43*, *KRAS* and *BRAF*, so no single molecular profile describes early-onset disease as a whole [43]. Separately, global hypomethylation increases as age at diagnosis falls. In 1356 tumours, mean methylation of long interspersed nucleotide element-1 declined from 64.7% in patients aged 70 years and over to 58.9% in those under 50, with an adjusted coefficient of minus 4.54 and an interval of minus 8.24 to minus 0.85 for the youngest group. However, that analysis rested on only 28 early-onset cases [44]. An earlier series of 343 tumours reported a mean methylation of 56.6% in cancers diagnosed at 50 years or younger, compared with 67.1% in sporadic microsatellite-unstable tumours, 65.1% in sporadic microsatellite-stable tumours, 66.3% in Lynch syndrome tumours, and 76.5% in normal mucosa [45]. Because this comparison separates early-onset disease from microsatellite-unstable and Lynch syndrome tumours, hypomethylation is not simply a consequence of either.

For this review, the molecular evidence supports three statements. For common driver mutations and microsatellite status, the early-onset disease profile differs from the later-onset profile in degree rather than kind, and global hypomethylation is the clearest candidate for a qualitative difference. The lower frequency of *BRAF* mutation and of the CpG island methylator phenotype points away from the serrated pathway as the route by which these tumours arise. None of the cited analyses measured molecular features against calendar time or birth cohort, so they describe early-onset tumours without accounting for why their number is increasing, which is also the conclusion the authors of the clinicogenomic series draw from their own data [4].

## 5. Modifiable Risk Factors

### 5.1. Obesity and Adiposity

Obesity is a broadly recognised risk factor for colorectal cancer [46,47]. Accumulation of visceral adipose tissue, which secretes adipokines that produce proinflammatory metabolites and cytokines, including IL-1β, IL-6, and TNF-α, promotes chronic inflammation. Moreover, both B and T lymphocytes infiltrate adipose tissue, further promoting local and systemic inflammation [48]. Additionally, lipid peroxidation in adipocytes generates reactive metabolites such as 4-hydroxy-2-nonenal (4-HNE) and malondialdehyde (MDA), which can contribute to colorectal carcinogenesis through oxidative damage and dysregulation of cellular signalling. 4-HNE can modify DNA, proteins, and lipids, thereby affecting mitochondrial function, DNA repair, and signalling pathways involved in cell survival and inflammation, including MAPK, PI3K/AKT, and PKC. MDA, in turn, exhibits potent mutagenic activity, thereby promoting genomic instability and facilitating tumour initiation and progression [49]. This interplay between chronic inflammation, immune dysregulation, and oxidative stress may promote colorectal carcinogenesis in the context of obesity. Furthermore, emerging evidence suggests that obesity-related gut dysbiosis might also play a role in the development of colorectal cancer [50,51]. Interestingly, the association between body mass index (BMI) and CRC risk appears consistent across molecular subtypes, including *KRAS*- and *BRAF*-defined tumours and CpG island methylator phenotype (CIMP) subgroups, with Lynch syndrome-associated CRC as a notable exception [52].

Many analyses are confirming the association between obesity and EOCRC. This association also extends to EOCRC. A systematic review by Hilbert et al. found that individuals with obesity at the time of diagnosis had a 1.45-fold higher risk of EOCRC. Furthermore, multiple cohort studies included in the review showed that elevated BMI in late adolescence (age 20) and at age 30 was associated with an increased risk of EOCRC, and that elevated BMI at these time points was an independent risk factor [53]. Consistent with these findings, a meta-analysis by Li et al. also demonstrated an increased risk of EOCRC among individuals with overweight or obesity, with the risk rising progressively with increasing BMI. Overweight was also identified as a risk factor, although the association was stronger among individuals with obesity [54].

Excess body weight, as measured by elevated BMI and visceral adiposity, has been associated with an increased risk of EOCRC, potentially mediated through several interconnected pathways. Excess adipose tissue induces chronic low-grade inflammation, leading to insulin resistance and hyperinsulinemia [55]. Moreover, obesity contributes to adverse changes in the gut microbiome. Together, these factors create a pro-tumorigenic microenvironment in the colon: immune signalling is disrupted, epithelial cell proliferation becomes uncontrolled, and natural apoptosis is impaired [55]. Regarding the association with earlier onset, exposure timing plays a crucial role. Early-life obesity (during childhood and adolescence) and prolonged obesity duration subject colorectal cells to continuous oxidative stress, inflammation, and hormonal stimuli [55]. This significantly shortens the time required for mutation accumulation and accelerates malignant transformation. Consequently, studies show a dose–response relationship: the longer elevated BMI and visceral adiposity persist, the higher the risk of developing EOCRC.

The mechanism by which older and younger patients develop EOCRC is the same. However, obesity remains a key factor explaining the divergent EOCRC trends because generational shifts change the timing and duration of exposure. Modern generations are exposed to excess adiposity much earlier in life and experience it for a larger proportion of their lifespan than older patients [55]. The increase in EOCRC incidence directly parallels the global rise in overweight and obesity among children and young adults [55].

### 5.2. Metabolic Dysfunction

Metabolic syndrome (MetS) includes central obesity, hypertension, and impaired glucose and lipid metabolism. The global prevalence of MetS has increased substantially over the past two decades [56]. Consequently, it is now a recognised risk factor for both average-onset CRC and EOCRC [57,58,59]. The risk of developing an early-onset malignancy scales directly with a patient’s number of metabolic comorbidities. Metabolic dysregulation actively drives colorectal carcinogenesis through insulin resistance, hyperinsulinemia, and altered insulin-like growth factor levels, particularly IGF-1 [60]. These hormonal shifts directly promote tumour growth and progression by altering the cellular environment [60]. In colorectal tissue, insulin and IGF-1 signalling activate the PI3K/AKT/mTOR and MAPK/ERK pathways [61]. This molecular cascade drives cellular proliferation, stimulates tumour angiogenesis and enhances epithelial cell migration, all while impairing natural apoptosis [60,61]. Furthermore, elevated circulating triglycerides and total cholesterol also significantly increase colorectal cancer risk [62]. When combined with gut microbiome disruption, these metabolic conditions fuel chronic low-grade inflammation, ultimately creating a highly pro-tumorigenic microenvironment [60]. Epidemiological data strongly reflect the impact of these metabolic disturbances on early-onset disease. EOCRC is associated with 43% higher odds of a diabetes diagnosis, with diabetes identified in 6% of patients [63]. Crucially, EOCRC risk depends heavily on clinical management. In the analysis, unmanaged diabetes was associated with elevated odds of EOCRC (OR 1.28, 95% CI 1.02–1.60), while managed diabetes was not (OR 1.04, 95% CI 0.84–1.28) [63]. However, without formal statistical interaction tests, current data cannot yet confirm whether actively managing diabetes directly reduces cancer risk. Metabolic dysfunction-driven biological mechanisms are not unique to young adults. Factors like chronic inflammation, hyperinsulinemia, and microbiome interactions promote carcinogenesis similarly in older patients. However, these conditions have distinct age- and sex-specific epidemiological patterns. Large database analyses reveal that the EOCRC risk associated with diabetes is strongest in the youngest cohorts, particularly among men aged 20–39 years [64]. Ultimately, while metabolic syndrome acts as a universal carcinogen, its rapid rise among younger generations is the critical differentiator. The early-life onset of these metabolic disorders accelerates cumulative tissue damage much sooner than in previous decades. Therefore, the modern epidemic of early-onset metabolic dysfunction drives trends in EOCRC incidence.

### 5.3. Dietary Patterns

Diet is an important factor influencing colorectal cancer risk. Mediterranean, DASH, vegetarian, and fasting diets may reduce CRC risk, whereas the Western diet is associated with higher CRC risk [65,66]. Western dietary patterns are characterised by low dietary fibre intake and high intake of processed and red meat. Western dietary patterns may also promote obesity, which in turn contributes to chronic low-grade inflammation and may further facilitate colorectal carcinogenesis [67].

The main benefits of dietary fibre include improved gut motility and reduced constipation, possible weight loss through reduced food intake, and potential improvements in insulin sensitivity and gut microbiota composition [68]. Moreover, low dietary fibre intake may contribute to low-grade chronic inflammation by impairing gut microbiota homeostasis and reducing the production of anti-inflammatory short-chain fatty acids, particularly butyrate [68]. High dietary fibre intake, particularly cereal fibre and whole grains, was associated with a reduced risk of colorectal cancer [69]. Therefore, insufficient fibre intake, a potentially protective dietary factor, could contribute to colorectal carcinogenesis.

Red meat consumption is a widely recognised risk factor for colorectal cancer [70,71]. The biological mechanisms linking red and processed meat consumption to colorectal carcinogenesis include the formation of N-nitroso compounds, lipid peroxidation, and the generation of heterocyclic amines and polycyclic aromatic hydrocarbons during high-temperature cooking, which may promote DNA damage, oxidative stress, and chronic inflammation. Additionally, microbiota-derived metabolites such as trimethylamine N-oxide (TMAO), generated from dietary carnitine and choline, may contribute to colorectal carcinogenesis through mechanisms involving oxidative stress and inflammation [70]. Notably, both fresh red meat and processed meat were independently associated with increased CRC risk, and risk increased progressively with higher consumption of red and processed meat, with the dose–response relationship remaining approximately linear up to around 140 g/day [71]. Other dietary factors, including high consumption of sugar-sweetened beverages, have also been associated with an increased risk of colorectal cancer [72], and a higher intake of ultra-processed foods has been associated with the risk of early-onset colorectal cancer precursors [73].

H_2_S has physiological functions in the gut; excessive or dysregulated H_2_S production may promote mucosal damage, inflammation and tumour-related signalling, suggesting a potential role in colorectal carcinogenesis [74]. Dietary patterns high in fat, protein, and sulfur promote sulfidogenic gut bacteria, leading to excessive microbial hydrogen sulfide (H_2_S) production. At elevated concentrations, this dysregulated H_2_S induces DNA damage, mucosal inflammation, and abnormal cellular proliferation, thereby actively driving colorectal carcinogenesis [75].

Western dietary patterns, along with red meat and sugar-sweetened beverage consumption, were associated with the development of EOCRC [66]. Moreover, higher adherence to the sulfur microbial diet was associated with an increased risk of early-onset adenomas, with the highest vs. lowest quartile associated with 31% higher odds (OR 1.31, 95% CI 1.10–1.56). The association was stronger for adenomas with greater malignant potential (OR 1.65, 95% CI 1.12–2.43), and similar trends were observed for dietary intake during adolescence [74]. Lower folate and calcium intake were also associated with higher EOCRC risk [76], whereas higher vitamin D intake (≥450 IU/day) was associated with lower EOCRC risk [77]. Importantly, dietary patterns may also influence the development of EOCRC precursors. Poor diet quality was associated with an increased risk of early-onset adenomas, particularly those with high malignant potential, suggesting that dietary factors may contribute to colorectal carcinogenesis at an early stage [78]. Interestingly, dietary patterns may differ between patients with early- and later-onset CRC. Compared with patients with older-onset CRC, those with EOCRC reported lower vegetable and fruit consumption and higher processed meat consumption, suggesting that dietary differences may contribute to the distinct epidemiology of EOCRC [79]. However, these differences may also reflect generational dietary patterns and should therefore be interpreted cautiously, warranting further investigation.

### 5.4. Ultra-Processed Foods

Ultra-processed foods (UPFs) are characterised by low levels of bioactive compounds such as fibre, vitamins, and polyphenols, and high levels of unhealthy fats, refined starches, and artificial additives [80,81]. Experimental models suggest these substances disrupt the gut microbiome, increase intestinal inflammation, and compromise mucosal integrity, thereby potentially influencing colorectal precursors [73]. In recent decades, the share of ultra-processed foods (UPF) in energy intake has risen by approximately 10%, meaning adults in the United States now derive nearly 60% of their total caloric intake from them [73]. A study by Wang et al. demonstrated that high consumption of ultra-processed foods significantly increases the risk of developing early-stage conventional adenomas in younger women, specifically those diagnosed before the age of 50. Mechanisms of action of ultra-processed foods (UPF), such as microbiome disruption, inflammation, and dietary toxins, can drive mutational processes that are significantly more common in younger patients [73]. UPFs likely drive EOCRC trends not because young people are uniquely vulnerable, but because recent generations have been exposed to much higher amounts of these foods since infancy. Mutations induced by colibactin-producing bacteria (pks^+^ *E. coli*) occur 2.5–4 times more frequently in EOCRC and permanently accelerate tumour development along the classic adenoma-carcinoma pathway [73,82]. It can also be more common in younger patients with EOCRC than in older ones. In contrast, the serrated pathway requires a longer timeframe, so UPFs have less impact on it in younger individuals [73]. Limiting products in this category is a key strategy for preventing colorectal neoplasia in the younger population.

### 5.5. Physical Inactivity

Physical inactivity and prolonged sedentary behaviour independently affect the risk of colorectal carcinogenesis. Extensive sedentary time significantly increases the risk of EOCRC. Individuals who sit 10 or more hours daily face nearly double the risk (OR 1.93) compared with those who sit under 5 h [83]. High television viewing volumes exceeding 14 h per week have been associated with similar outcomes (RR 1.69) [84]. Interestingly, sedentary-associated EOCRC shows a strong predilection for the rectum and distal colon [83,85]. This shows inflammatory patterns seen in colitis. It suggests that physical inactivity may induce region-specific alterations in the gut microbiome and local immune responses. The absence of movement extends the contact duration between the colonic mucosa and faecal carcinogens, such as secondary bile acids [60]. Sedentary behaviour also promotes abdominal adiposity and alters the gut microbiome (dysbiosis), creating a highly pro-tumorigenic metabolic environment. Prolonged sitting lowers vitamin D levels [85]. This risk remains elevated even in individuals who engage in regular physical activity, which suggests that prolonged sitting contributes independently of physical activity.

In contrast, the role of physical activity remains inconsistent. Some analyses link regular moderate or vigorous exercise to a significant reduction in EOCRC risk [85]. Several smaller cohort studies reported no significant effect [84,86]. This highlights active sedentary behaviour as a more prominent risk factor than not exercising.

The physiological pathways connecting a sedentary lifestyle to cancer can be found across all age groups 20–49. Extended daily sitting time strongly correlates with elevated EOCRC rates, suggesting that prolonged sedentary time is an independent risk factor associated with early-onset tumour development [83]. Modern youth engage in more sedentary behaviour. Increased screen time and desk work fundamentally reduce daily energy output. The risk is greater in overweight individuals [85]. Older age groups had higher physical activity levels during their early decades. The carcinogenic impact of sedentary time is being spread evenly in young individuals without a family history of colorectal cancer (RR 1.83) [85].

### 5.6. Alcohol

Alcohol is a well-established carcinogen, although its specific association with the EOCRC remains inconsistent across epidemiological studies [87]. The carcinogenic effects of alcohol are known to be modulated by complex interactions with individual genetic susceptibility and other lifestyle factors. Regarding the early age onset, prospective data demonstrate a dose–response relationship, indicating that moderate and heavy alcohol consumption contributes to the increased risk of EOCRC [88,89]. On the other hand, Glover et al. examined young adults aged 20–39 and found no significant association between alcohol use and EOCRC risk [90]. Studies have also shown that moderate drinking (10–20 g/day for women, 10–30 g/day for men) and heavy drinking (≥20 g/day for women, ≥30 g/day for men) both elevate EOCRC risk, with these associations often appearing significantly stronger in men [91]. Two smaller studies have shown no significant association between alcohol use and EOCRC [57]. Alcohol may promote early-onset carcinogenesis through dysbiosis and epigenetic alterations like LINE-1 hypomethylation. Epidemiological evidence linking it to EOCRC remains highly heterogeneous. Explaining population-level trends requires distinct generational shifts in exposure rather than unique age-specific mechanisms. Current data is insufficient to identify alcohol as a primary cause of the recent birth-cohort increases in EOCRC [86].

### 5.7. Tobacco

Similarly to alcohol, tobacco is also a well-established carcinogen. However, its contribution to EOCRC development has been inconsistent across studies. Large registry-based analyses report an increased risk of EOCRC among smokers. For early-onset EOCRC, risk appears to be influenced by cumulative exposure. The cohort analyses demonstrate an increased risk found among current heavy smokers (≥10 pack-years) for all ages in men (20–39 and 40–49) and women aged 40–49. At the same time, light or former smokers show no significant associations for women aged 20–30 years. On the other hand, several smaller case–control and cross-sectional studies reported no significant association between smoking and EOCRC risk [57].

The carcinogenic impact of tobacco can be found in both young and older adults, with no unique biological mechanism specific to early-onset disease. The epidemiological evidence linking tobacco exposure to EOCRC is heterogeneous and inconsistent. Hence, while tobacco induces the same biological effect regardless of age, its epidemiological impact differs by sex. The risk of EOCRC increases across male cohorts; however, among women it is limited to those aged 40–49 years, with no significant effect observed in younger females. Therefore, tobacco exposure cannot fully explain the rising EOCRC incidence trends, particularly in younger female cohorts [57].

### 5.8. Antibiotic Exposure and Gut Microbiome

Medication exposure, particularly long-term or recurrent antibiotic use (LRAU) during childhood or adolescence, can cause severe and long-standing disruption of the gut microbiome [92,93]. EOCRC is linked to distinct intestinal microbial features which include enrichment of proinflammatory and genotoxic bacteria such as Fusobacterium nucleatum, colibactin-producing *E. coli*, and enterotoxigenic Bacteroides fragilis [94]. These microbial imbalances allow pathogenic bacteria to colonise and invade the gut mucosa, impair immune surveillance, and trigger chronic inflammation that causes direct genotoxic damage and early tumour initiation. Regarding the relationship with an early age of onset, early-life antibiotic exposure establishes a long-lasting dysbiosis that is hypothesized to contribute to colorectal carcinogenesis. It shows a significantly increased risk of early-onset adenomas and EOCRC. This early-onset risk is particularly pronounced when childhood or adolescent antibiotic use interacts with host genetic susceptibilities, such as high polygenic risk scores, a family history of colorectal cancer, or specific variations in the *FUT2* gene (e.g., rs281377 TT/CT genotypes), which regulate gut microbiota composition [95].

While antibiotic-induced dysbiosis can affect individuals of any age, biological vulnerability and epidemiological impact differ depending on the timing of exposure. Antibiotic usage in early life may increase the risk of EOCRC, though this primarily affects patients with a genetic predisposition and a family history of the disease [92]. The risk of adenoma development rises with antibiotic therapy regardless of overall genetic predisposition; however, it is mainly found in patients carrying the TT/CT variant of the *FUT2* gene. These differences suggest that antibiotics interact with distinct genetic mechanisms depending on the stage of carcinogenesis, promoting the initiation of adenomas through *FUT2*, while contributing to the development of EOCRC in individuals with high overall genetic predisposition. Mutations in this gene can disrupt the gut microbiome, triggering inflammation and damaging the intestinal epithelial barrier. Microbiome disruption during the critical developmental years of childhood or adolescence often leads to incomplete microbial recovery. These long-standing, early-life alterations differ fundamentally from dysbiosis acquired in adulthood.

Furthermore, because modern generations have experienced a widespread increase in LRAU for childhood infections compared to older cohorts, this early-life environmental shift represents a unique exposure. Therefore, childhood LRAU helps explain the rising incidence of EOCRC in younger generations [92]. While early antibiotic exposure alone may slightly increase cancer risk, the danger can be significantly greater in individuals who carry specific genetic traits (such as *FUT2* mutations) that weaken the gut microbiome. This combination of childhood medication and genetic vulnerability causes lasting dysbiosis, creating a gut environment that encourages tumour development [92]. These findings highlight the role of early-life microbiome disruption in carcinogenesis. However, further research is needed to determine which antibiotics, and for how long, pose the greatest risk. However, studies involving older patients link the risk of bowel cancer primarily to broad-spectrum antibiotics. Because the UK Biobank lacks detailed data, it is harder to examine this association in the context of early-onset colorectal cancer (EOCRC) [92]. Further research into specific types of antibiotics is essential to develop safer treatment guidelines for children with a high genetic risk.

### 5.9. Inflammatory Conditions

Inflammatory bowel diseases (IBD) are a heterogeneous group of disorders such as ulcerative colitis and Crohn’s disease, which subject the intestinal epithelium to continuous cycles of injury and repair [96,97]. Recurrent mucosal injury and repair promote genetic and epigenetic alterations that drive the progression of normal epithelium toward dysplasia and neoplasia. These diseases affect individuals across all age groups and sexes and present with variable clinical manifestations.

Chronic intestinal inflammation causes the accumulation of harmful oxygen species, leading to oxidative stress. This state rapidly damages cells and triggers mutations, for instance, by inactivating the p53 gene, which normally protects against cancer-this damage is found in 83% of cancer patients with IBD [96,98]. In IBD patients, key mutations (such as *APC* mutations) appear very late in the disease, unlike in sporadic adenoma-carcinoma [98]. Chronic inflammatory factors such as TNF trigger signalling pathways in epithelial cells that prevent their natural death [96,98]. As a result, the immune system becomes imbalanced: cells that drive inflammation (Th1, Th17) increase, while cells that resolve inflammation decline. If this state persists, damaged cells that resist death can develop dysplasia [96].

The risk of tumour development strongly correlates with the duration of intestinal inflammation. Based on a 2001 meta-analysis of 116 studies from 1966 to 1999, the cumulative risk of colorectal cancer specifically among patients with ulcerative colitis (UC) was estimated at 2% after 10 years, 8% after 20 years, and up to 18% after 30 years of disease duration [98]. The age at onset of inflammatory bowel disease drastically alters the risk of developing cancer. For inflammatory bowel disease (IBD) generally, a nationwide Danish cohort study conducted between 1979 and 2008 demonstrated that individuals diagnosed in childhood or adolescence ages 0–19 faced a relative risk of developing CRC nearly 44 times higher than the general population, compared to a 2.65-fold increase for those diagnosed between the ages of 20 and 39 [98]. These estimates derive primarily from historical cohorts and may significantly overestimate the risk for contemporary patients. Recent epidemiological data suggest a declining incidence of IBD-related CRC in modern clinical practice [98]. This reduction is being shown by the widespread use of chronic maintenance therapies, including immunomodulators, anti-TNF and anti-integrin agents. Those therapies promote mucosal healing, alongside enhanced colonoscopic surveillance programs which enable the early detection and resection of dysplasia.

Due to ongoing cell damage and inflammation, colorectal cancer in patients with IBD is diagnosed on average 7.7 years earlier than non-inflammation-related cancers [99]. Due to this temporal factor, cancers in young adults very often have an inflammatory basis among cancer patients under the age of 30. One in three cases was linked to predisposing conditions, including genetic factors and IBD [99]. As many as 20% of early IBD-associated cancers are highly aggressive mucinous and signet-ring cell tumours, and nearly 40% of them present with metastases at the time of diagnosis [99]. The mechanism by which years of inflammation and associated damage contribute to cancer risk appears similar across all age groups. The fact that patients develop cancer at an early age for this reason is due to the disease’s onset in early youth, which consequently reaches the threshold of earlier exposure to inflammation [98].

### 5.10. Environmental Exposures

Under the exposome theory, early-life pesticide exposure may promote carcinogenesis by disrupting the gut microbiome [100,101]. For instance, aquatic pesticide pollution might explain the higher EOCRC risk seen in frequent fish consumers [102]. However, direct epidemiological evidence linking pesticides to EOCRC remains limited [57]. Most current data are observational and derived from late-onset colorectal cancer [57]. Early-life pesticide exposure increases individual cancer risk. However, this does not explain the population-wide rise in EOCRC. Current data is lacking. Therefore, pesticides cannot fully explain the rising EOCRC trends in comparison to late-onset colorectal cancer.

PFAS are persistent synthetic chemicals that accumulate in human tissues [103]. Their specific impact on colorectal cancer remains less defined [103]. At the cellular level, compounds like PFOA and PFOS stimulate colorectal cancer cell proliferation and migration [104,105,106]. They activate the PI3K/Akt-NF-κB pathway, induce epithelial–mesenchymal transition and upregulate MMP-2 and MMP-9 enzymes [104,105,106]. In population studies, high exposure correlates with more gastrointestinal cancers, gut dysbiosis and altered bile acid metabolism [107]. Additionally, PFOS exposure downregulates the ketogenic enzyme HMGCS2 in intestinal cells and organoids [108,109]. PFAS also exert immunotoxic effects by impairing antibody production and T-cell function [107]. This impairs the immune clearance of early neoplastic lesions [107]. Biologically, these oncogenic and immunosuppressive mechanisms operate similarly across all ages. However, PFAS may explain divergent generational trends through a birth-cohort effect. Large-scale industrial use of PFAS began in the mid-20th century. This aligns with the rise in incidence observed among individuals born after 1950 [103].

Microplastics (MPs) damage the mucosal barrier by reducing *MUC2* gene expression. This allows *E. coli* to penetrate the compromised layer and directly contact epithelial cells. Lifelong accumulation of MPs since infancy may be the cause of early-onset colorectal cancer in younger generations [110]. However, direct human epidemiological studies associating microplastic accumulation with invasive EOCRC risk have not yet been conducted. While it has been hypothesized that lifelong accumulation of MPs could be an emerging risk factor, direct epidemiological evidence linking microplastics to EOCRC in humans is currently lacking. Therefore, their potential role in driving current incidence trends remains speculative and requires cautious interpretation. Delivery by Caesarean section drastically disrupts the transfer of the maternal microbiome and is a significant risk factor for early-childhood gut dysbiosis. This leads to delayed colonisation by *Bifidobacterium* species, which normally exert protective, anticancer properties and assist in infant immune education [111]. Because early infancy is a critical period for immune system maturation, this altered flora can lead to immune deficiencies or abnormal immune development [112]. Caesarean section is also associated with an increased risk of obesity, insulin resistance and diabetes. These conditions, which can appear later in life, may increase the risk of EOCRC [113,114]. The early age of onset stems from birth disruptions that affect the body throughout life. Consequently, it should be interpreted with caution, since there has been limited epidemiological evidence.

This effect is considered specific to the development of early-onset cancer, as it involves perinatal exposure and early-childhood microbiome disruption. One retrospective analysis observed an elevated risk of EOCRC among women delivered by Caesarean section (aOR 1.62), but found no association among men or in the cohort overall [115]. Because no subgroup interaction was demonstrated and the overall association was non-significant, these findings should be regarded as hypothesis rather than evidence of a sex-specific population trend. Overall, the individuals who were born through cesarean delivery did not have significantly higher odds of an EOCRC [115].

**Figure 3:**
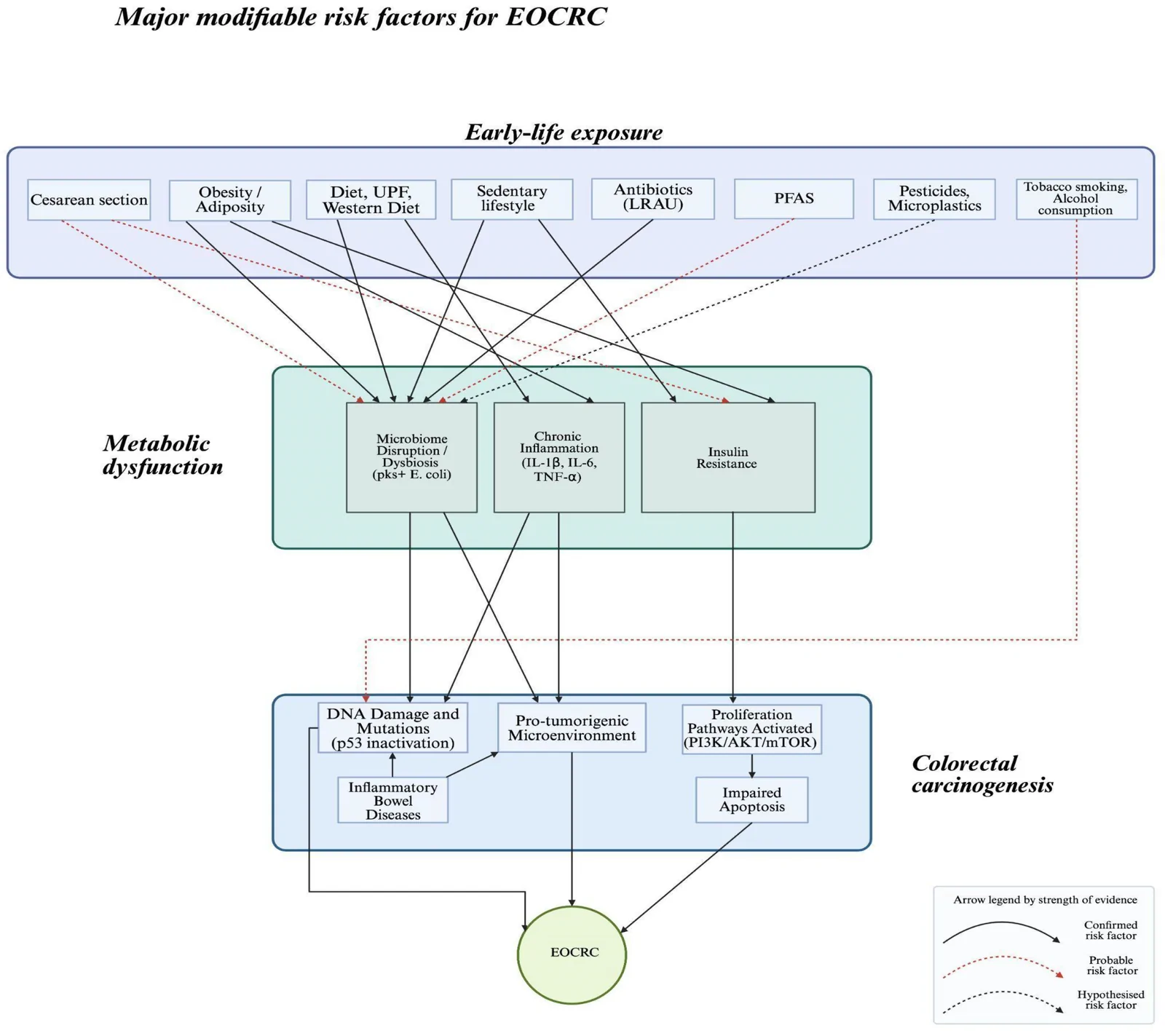
Major modifiable risk factors for EOCRC and their pathways. The arrows indicate the strength of evidence linking early-life exposures to metabolic dysfunction and colorectal carcinogenesis. Established risk factors (confirmed, solid black arrows, such as [82]). Emerging risk factors are categorised as either probable (dashed red arrows, such as [111]), indicating growing but limited evidence or hypothesised (dotted black arrows, such as [110]), based primarily on experimental models.

Figure 3 summarises the exposures discussed in this section and the pathways through which they are proposed to act.

## 6. From Risk Factors to Population-Level Incidence

Section 5 established that several modifiable factors, including obesity, physical inactivity and unhealthy dietary patterns, are associated with colorectal cancer risk. However, identifying individual risk factors alone does not explain why EOCRC incidence has increased at the population level. To address this question, we need to consider how exposure to these factors has changed across populations and generations, when such exposures occur, and whether they tend to cluster within individuals.

### 6.1. Population-Level Trends in Obesity and EOCRC

The prevalence of overweight and obesity has increased substantially worldwide over the past decades, affecting both adults and younger generations. Between 1975 and 2016, global age-standardized obesity prevalence among children and adolescents increased from 0.7% to 5.6% in girls and from 0.9% to 7.8% in boys, while adult obesity prevalence has continued to rise in many countries, reaching 42.8% in the United States in 2017–2018 [116]. Although obesity trends have plateaued in some high-income populations, the global obesity prevalence is rising, possibly due to increases in low-income populations [117]. This is particularly relevant to EOCRC incidence, given the established association between obesity and EOCRC risk discussed in Section 5.1. This raises the possibility that increasing population exposure to obesity may have contributed to the observed rise in EOCRC incidence.

At the population level, the increasing prevalence of obesity has coincided with rising EOCRC incidence. Across multiple countries, increases in overweight and obesity were positively correlated with the incidence of early-onset obesity-related cancers, including colorectal cancer [118]. Similarly, Chen et al. reported a positive correlation between increases in overweight and obesity prevalence and early-onset rectal cancer incidence at a 15-year lag. However, the association for colon cancer did not reach statistical significance [119]. Together, these findings suggest that the rise in obesity may be relevant to the disproportionately increasing incidence of colorectal cancer among younger adults. However, the observed associations vary by cancer subsite and do not establish causality. The burden of EOCRC attributable to high BMI has also increased globally, with a consistently greater and more rapidly increasing burden observed among men [120].

Taken together, the parallel increase in obesity prevalence and EOCRC incidence, alongside the growing global burden of EOCRC attributable to high BMI, supports the hypothesis that increasing population exposure to excess adiposity may have contributed to the rise in EOCRC. However, these population-level trends do not establish causality, particularly given substantial regional variation in EOCRC incidence and the influence of demographic factors on absolute case numbers. The observed increase in EOCRC likely reflects the combined effects of changing obesity prevalence and other population-level exposures rather than a single risk factor.

### 6.2. Age-Specific Exposure Patterns

While increasing population exposure to obesity may have contributed to the rise in EOCRC incidence, exposure timing may also matter. Because EOCRC develops at a relatively young age, exposure to excess adiposity during childhood, adolescence, or early adulthood may be relevant when considering the timing and duration of obesity-related risk. Importantly, the increasing prevalence of obesity is also evident among younger populations, with the prevalence of obesity in children and adolescents increasing 1.5-fold in 2012–2023 compared with 2000–2011 [121].

The risk of EOCRC may depend not only on obesity but also on the timing and persistence of excess adiposity. Although evidence specifically linking childhood obesity to EOCRC remains limited, studies of CRC overall suggest that higher body fatness during childhood and adolescence may contribute to later colorectal carcinogenesis [122]. Obesity during early adulthood has been associated with a substantial increase in EOCRC risk, with obesity at age 20 and 30 years associated with 2.56-fold and 2.06-fold higher risks, respectively, compared with BMI < 25 kg/m^2^ [123]. Similarly, among women, higher BMI at 18 years of age was associated with subsequent EOCRC risk. Compared with women with a BMI of 18.5 to 20.9 at 18 years of age, the RR of EOCRC was 1.32 (95% CI, 0.80–2.16) for women with a BMI of 21.0 to 22.9 and 1.63 (95% CI, 1.01–2.61) for women with a BMI of 23.0 or greater, although the pattern across BMI categories was not clearly linear (*p* = 0.66 for trend) [18]. Therefore, higher BMI in early adulthood may be relevant to EOCRC risk across a broader range of BMI values. However, the evidence for an association within the normal BMI range is less consistent. Collectively, these findings suggest that adolescence and early adulthood may represent particularly relevant periods during which excess adiposity may influence EOCRC development.

Beyond the age at which obesity develops, the persistence and trajectory of excess adiposity may also be relevant to EOCRC risk. In a large Korean nationwide cohort of more than 3.3 million individuals younger than 50 years, persistent obesity and persistent abdominal obesity were associated with an increased risk of EOCRC compared with individuals who remained non-obese (HR 1.09, 95% CI 1.03–1.16 and HR 1.18, 95% CI 1.09–1.29, respectively) [124]. Similarly, among women, greater weight gain since early adulthood was associated with progressively higher EOCRC risk, with a 9% increase in risk for each 5 kg increase in body weight and more than a two-fold risk among those who gained ≥ 40 kg since the age of 18 years [18]. These findings suggest that cumulative exposure to excess adiposity and unfavourable weight trajectories beginning in early adulthood may be particularly relevant to EOCRC risk.

Further longitudinal studies are needed to determine whether earlier and longer exposure confers greater risk.

### 6.3. Co-Occurrence and Cumulative Effects of Risk Factors

Risk factors for EOCRC may frequently occur in combination rather than in isolation. Obesity, physical inactivity, unhealthy dietary patterns and elements of metabolic syndrome often coexist within individuals and populations, raising the possibility that their combined effects may contribute to a greater risk of EOCRC than exposure to any single factor alone. However, because these risk factors often overlap, determining their independent effects is challenging.

A systematic review and meta-analysis by Hua et al. identified numerous individual risk factors associated with EOCRC, including obesity, sedentary lifestyle, Western dietary patterns, alcohol consumption, smoking, and metabolic syndrome [58]. However, the substantial overlap between these exposures makes it difficult to determine their independent and potentially interacting contributions to EOCRC risk. Similarly, a recent pooled analysis of three European cohorts, which defined EOCRC as CRC diagnosed before age 55 years, identified several metabolic and lifestyle factors associated with early-onset disease. However, they did not assess their combined effects [2]. A recent systematic review and meta-analysis by Hilbert et al. found that several metabolic disorders, including type 2 diabetes, hyperlipidemia, hypertension, and metabolic syndrome, were associated with an increased risk of EOCRC. Importantly, EOCRC risk increased with more coexisting metabolic disorders, suggesting that the cumulative burden of metabolic dysfunction may matter more for EOCRC risk than individual metabolic risk factors alone [53]. Similarly, Jin et al. reported a dose–response relationship between the number of metabolic syndrome components and EOCRC risk [125]. Lundqvist et al. examined the potential interaction between metabolic and autoimmune diseases, finding that the association between IBD and EOCRC differed according to the presence of metabolic disease. Although IBD was strongly associated with EOCRC both in individuals with and without metabolic disease, the observed association was stronger among those without metabolic disease (HR 7.52, 95% CI 5.62–10.06) than among those with metabolic disease (HR 3.65, 95% CI 2.57–5.19) [126]. These findings suggest that the association between one risk factor and EOCRC may vary according to the presence of other coexisting conditions, although the underlying mechanisms remain uncertain.

The gut microbiota may represent one potential biological pathway through which multiple coexisting exposures may converge on mechanisms relevant to colorectal carcinogenesis [94,127]. Obesity, dietary patterns and metabolic dysfunction can all influence the intestinal microbial environment, raising the possibility that persistent or cumulative alterations in the gut microbiota may represent one mechanism through which these exposures could jointly influence colorectal carcinogenesis [86,127]. However, whether microbiota-related changes mediate or modify the joint effects of metabolic and lifestyle risk factors in EOCRC remains insufficiently understood.

Overall, current evidence suggests that cumulative metabolic dysfunction may be relevant to EOCRC risk, whereas evidence for interactions or synergistic effects between broader lifestyle factors remains limited. Further research is needed to clarify how coexisting risk factors jointly influence EOCRC risk.

## 7. Future Burden of Early-Onset Colorectal Cancer

The epidemiological patterns described above raise an important question: how will the burden of EOCRC evolve in the coming decades? The observed increase in incidence, its concentration in younger birth cohorts, and the substantial geographic heterogeneity of these trends suggest that the future burden of EOCRC is unlikely to be distributed uniformly across populations [11,128]. At the same time, factors associated with EOCRC, including obesity and metabolic dysfunction, dietary patterns, physical inactivity, alcohol and tobacco exposure, inflammatory conditions, and potentially early-life environmental and microbiome-related exposures, are themselves changing across generations [11,25,129]. The future trajectory of EOCRC will therefore reflect the combined effects of demographic change, evolving exposure patterns, changes in diagnostic practices, and the persistence or modification of cohort-specific risk [11,17,128,130].

The available evidence suggests that these trajectories are already diverging between populations [11,128,131]. Some high-income countries that experienced prolonged increases in EOCRC incidence have begun to show stabilization in selected age groups, while increasing trends are emerging in several populations in Eastern Europe, Asia, and Latin America and the Caribbean [128,131,132]. At the same time, rapidly changing patterns of obesity, metabolic dysfunction, diet, sedentary behaviour, and other early-life exposures may influence incidence in successive generations [11,25,129]. These developments make it difficult to assume that historical trends will continue unchanged.

### 7.1. Incidence Projections

Near-term projections consistently indicate continued growth in EOCRC incidence through approximately 2030. Using Global Burden of Disease (GBD) 2021 data and a Bayesian age–period–cohort (BAPC) model, Meng et al. projected an increase in the global age-standardized incidence rate (ASIR) from 6.13 per 100,000 in 2021 to approximately 6.46 per 100,000 in 2030 [128]. Autoregressive integrated moving average (ARIMA)-based forecasts point in the same direction: Zhang et al. projected a 5.56% increase in global EOCRC ASIR between 2021 and 2036 [25], whereas Nie et al. estimated that incidence would reach approximately 6.70 per 100,000 by 2031 [129]. Despite differences in magnitude, these models broadly indicate continued near-term growth in ASIR [25,128,129].

By approximately 2040, the projected picture becomes less uniform. Using a Nordpred age–period–cohort model, Pan et al. projected approximately 270,800 new EOCRC cases, 1.77 million prevalent cases, 90,700 deaths, and 4.54 million DALYs globally in 2040 [20]. However, age-standardized incidence and prevalence were projected to increase only until about 2034, then decline [20]. Uncertainty increases further toward 2050. Li et al. projected that EOCRC deaths and DALYs would fluctuate but generally increase, reaching approximately 85,600 deaths and 4.28 million DALYs globally by 2050 [133]. However, these estimates describe mortality and disability rather than the corresponding incidence trajectory. Tawfiles et al. estimated approximately 226,700 new EOCRC cases and 94,400 deaths globally in 2022. They projected further increases in incidence and mortality toward 2050, with particularly large future burdens in low-HDI countries, African regions, and South-Central Asia [131]. Importantly, interpret these mortality estimates within their respective modelling frameworks rather than as successive points in a single temporal trajectory. The differences in data sources, modelling methods, baseline years, and underlying assumptions preclude direct comparison of the reported estimates and therefore do not support interpreting the values of 94,400 deaths in 2022, 90,700 in 2040, and 85,600 in 2050 as evidence of declining global EOCRC mortality over time.

Table 1 summarises the principal characteristics, assumptions, and limitations of these projection models.

Overall, projections more consistently indicate continued growth in the absolute burden of EOCRC than they do in defining the long-term direction of global age-standardised incidence [20,128,131,133]. Models generally agree on continued near-term growth [25,128,129], whereas some longer-term projections suggest that ASIR may plateau or decline during the 2030s; the absolute number of EOCRC cases is expected to continue increasing [20]. These divergent trajectories reflect how demographic change shapes the future burden of disease. ASIR accounts for differences in population age structure and therefore primarily reflects changes in underlying incidence risk. In contrast, the absolute number of cases also reflects population size and demographic composition. Consequently, even a stable or declining ASIR does not necessarily reduce the number of individuals diagnosed with EOCRC, particularly in settings experiencing population growth or expansion of the age groups at risk. This distinction matters for interpreting long-term global projections, where changes in population structure can substantially alter the absolute burden despite relatively modest changes in incidence rates. No single consensus trajectory for global EOCRC ASIR to 2050 has therefore been established [20,25,128,129,131,133].

### 7.2. Why the Projections Disagree

The projections are not directly comparable because studies estimate different outcomes and use varying EOCRC definitions, age ranges, standard populations, and historical periods for model fitting [20,25,128,131,133].

This is apparent even among GBD-based analyses. For 2021, Meng et al. reported a global EOCRC ASIR of approximately 6.13 per 100,000 [128], whereas Zhang et al. reported 5.37 per 100,000 [25] and Pan et al. reported approximately 5.15 per 100,000 [20]. These differences partly reflect non-identical age ranges and age groupings; because CRC incidence rises across young adulthood, the population included in each analysis affects its baseline estimates and subsequent projections [128,134].

Model structure represents a second major source of divergence. BAPC models explicitly estimate age, calendar-period, and birth-cohort components [128]. ARIMA models primarily extrapolate the temporal dependence present in the observed time series [25]. In contrast, Nordpred age–period–cohort models attenuate long-term drift rather than assuming that recent trends will continue indefinitely [20]. Consequently, different modelling approaches may produce broadly similar short-term forecasts but increasingly divergent trajectories as the projection horizon extends [20,25,128].

Birth-cohort effects add another source of uncertainty. As Section 3.2.1 describes, EOCRC risk is not distributed uniformly across successive generations. Age–period–cohort analyses from Australia, Canada, England, and the United States show increasing EOCRC risk across successive birth cohorts, with individuals born in the 1990s facing substantially higher risk than those born in the 1960s [11]. For forecasting, the critical issue is that these cohorts move through, and eventually leave, the age range that defines EOCRC. For example, individuals born in 1990 contribute to EOCRC incidence only until they reach age 50 in 2040. We cannot assume that younger cohorts with similar or greater risk offset their departure. Long-range forecasts that primarily extrapolate calendar-period trends may therefore misestimate incidence if they do not adequately represent cohort turnover [11].

Historical incidence series may also contain structural changes related to screening, diagnostic intensity, tumour classification, and cancer registration. Changes in histological classification and registration can substantially alter measured EOCRC trends without necessarily representing equivalent changes in the underlying incidence of colorectal adenocarcinoma [17]. Similarly, diagnostic and colonoscopy use can affect observed incidence, although evidence indicates that increasing colonoscopy use alone does not explain the rise in EOCRC [130]. Consequently, extrapolating a temporary increase in detection could overestimate future incidence, whereas a model fitted to an earlier diagnostic period could underestimate recorded incidence after lasting changes in practice.

Finally, most statistical forecasts extrapolate historical incidence patterns according to the assumptions of the selected model rather than dynamically modelling future changes in risk factors, screening uptake, or preventive interventions [20,25,128]. Scenario-based projections therefore answer a different question from conventional trend extrapolation: they estimate burden conditional on a specified future pathway rather than predicting a single inevitable outcome.

### 7.3. Age-Specific and Sex-Specific Projections

Age-specific projections distinguish groups expected to contribute many cases from those with the steepest relative increases. In a BAPC analysis restricted to adults aged 40–49 years, the global ASIR was projected to reach approximately 18.42 per 100,000 in 2030, representing a 20.1% increase from 2019 [135]. The relatively high incidence in this age group suggests that adults aged 40–49 years will continue to account for a substantial share of near-term cases.

The same projection demonstrates that age-specific burden must be interpreted separately from the rate of change. Bailey et al. estimated that between 2010 and 2030 colon and rectal cancer incidence in the United States would increase by approximately 90% and 124%, respectively, among adults aged 20–34 years, compared with increases of 28% and 46% among those aged 35–49 years [134]. Thus, the youngest adults may experience the steepest relative increases while still contributing fewer absolute cases than individuals closer to age 50.

The longer-term age distribution is more difficult to predict because it depends on whether elevated risk persists as younger birth cohorts age. Cohorts contributing relatively few cases while in their 20s or 30s may generate substantially greater absolute burden when they enter their 40s, when baseline CRC incidence is much higher. Conversely, those cohorts eventually leave the EOCRC definition after age 49. Consequently, future age-specific burden depends not only on the rate of increase within each age group but also on how successive cohorts move through the fixed <50-year age boundary. Long-term forecasts must therefore account for both cohort ageing and the eventual exit of these cohorts from the under-50 population.

Sex-specific projections also suggest persistent heterogeneity. Meng et al. reported a 2021 ASIR of 7.28 per 100,000 in males compared with 4.96 per 100,000 in females and projected that the greater male burden would persist through 2030 [128]. Zhang et al. similarly projected male incidence to reach approximately 6.72 per 100,000 by 2036, representing a 10.3% increase, whereas the female rate was projected to remain comparatively stable at approximately 4.16 per 100,000 [25].

Age-specific modelling of adults aged 40–49 years provides further evidence that this difference may persist in the near term. By 2030, the projected incidence ASR in this age group was approximately 23.42 per 100,000 in males compared with 13.43 per 100,000 in females, corresponding to increases of 27.4% and 9.7%, respectively [135]. Long-term BAPC projections for the BRICS countries likewise indicate persistent sex disparities through 2050, although their magnitude differs between countries [132].

Overall, adults aged 40–49 years are projected to account for a substantial share of near-term cases, while younger adults may experience steeper relative increases as higher-risk cohorts age. Males are projected to have higher incidence in many settings, although the magnitude of the sex difference varies by population [25,128,132,134,135].

### 7.4. Geographic Projections

Future geographic patterns will reflect both epidemiological trends and demographic change; regions with the highest age-standardised incidence will not necessarily experience the largest increases in absolute case numbers.

Available long-range projections suggest these two dimensions of burden may diverge further. Tawfiles et al. projected substantial growth in EOCRC cases and deaths through 2050, with particularly large increases in low-HDI countries, African regions, and South Central Asia [131]. Projections for the BRICS countries illustrate how heterogeneous these future trajectories may be. BAPC modelling to 2050 indicates that India and South Africa are likely to experience some of the largest increases in EOCRC burden, while China is projected to maintain a high absolute burden; Russia, in contrast, is expected to remain comparatively stable or decline [132]. Thus, no single geographic pathway emerges, even among large middle-income countries. Similar future increases in case numbers may also result from different mechanisms: decomposition analysis identified population growth as the predominant driver of increasing incidence burden in India, whereas epidemiological change contributed particularly strongly in South Africa [132].

Uncertainty around projections is also uneven across regions. Long-range estimates for regions with incomplete population-based cancer registration or limited diagnostic infrastructure begin from less certain baseline incidence estimates. This is particularly relevant in parts of sub-Saharan Africa, where registry coverage and diagnostic capacity remain limited [136]. Improvements in ascertainment over coming decades could therefore increase recorded incidence independently of, or in addition to, genuine epidemiological change. Forecasts for such settings may therefore be less certain than projections based on long-running population-based registries.

These patterns suggest a possible redistribution of the global EOCRC burden rather than a uniform worldwide increase. High-income regions may continue to carry relatively high age-standardised incidence, whereas rapidly growing populations in South Asia, Africa, and other transitioning settings may contribute an increasing proportion of future absolute cases [131,132]. Therefore, which regions experience the greatest healthcare burden will depend not only on the trajectory of EOCRC risk but also on the size and age structure of the population exposed to that risk.

Overall, projections suggest a potential redistribution of EOCRC burden rather than a uniform worldwide increase. Future regional healthcare demand will depend on incidence trajectories, demographic change, and the reliability of baseline cancer registration [20,128,131,132,136].

## 8. Implications for Prevention and Screening

The increasing exposure of younger generations to obesity, metabolic dysfunction, unhealthy dietary patterns, physical inactivity, and other potentially modifiable factors highlights the need to consider colorectal cancer prevention across the life course rather than exclusively within the age range traditionally targeted by population-based screening. Evidence in the preceding sections indicates that several EOCRC risk factors may be established during childhood, adolescence, or early adulthood, potentially increasing cumulative exposure to metabolic, inflammatory, and environmental processes involved in colorectal carcinogenesis [57,80].

At the same time, EOCRC does not represent a uniform risk category. The burden of disease reflects the interaction between potentially modifiable exposures and established predisposing conditions, including a family history of colorectal cancer and inflammatory bowel disease. This raises important questions about whether prevention and surveillance strategies should be adapted to individual risk profiles rather than applied solely based on chronological age [80,137].

Accordingly, EOCRC prevention strategies require a multidimensional approach that includes primary prevention of modifiable risk factors, risk-stratified preventive interventions, and strategies to detect premalignant or early malignant lesions before symptomatic presentation [57,80,137].

### 8.1. Primary Prevention

Primary prevention is particularly relevant to EOCRC because potentially important exposures may begin years or even decades before the age at which population screening is usually offered. Consequently, prevention cannot be limited to the period immediately preceding screening eligibility but should instead address modifiable exposures throughout the life course [80,138]. At the same time, most EOCRC-specific evidence is derived from observational studies, and associations between individual exposures and EOCRC do not establish that modification of any single factor will independently reduce EOCRC incidence [57]. Evidence linking modifiable and established predisposing factors to EOCRC supports prevention strategies across the life course, including population-level interventions and risk-adapted approaches [57,80,138].

Given the associations described in Section 5, maintaining a healthy body weight and metabolic health is an important primary prevention target. Excess adiposity and metabolic dysfunction often develop during childhood, adolescence, or young adulthood. They may therefore prolong exposure to metabolic and inflammatory abnormalities before EOCRC develops [53,57,80]. A 2026 systematic review and meta-analysis found a 1.45-fold higher risk of EOCRC among individuals with obesity, while longitudinal evidence linked elevated BMI in late adolescence and at ages 20 and 30, persistent obesity, and abdominal adiposity with increased EOCRC risk. Metabolic syndrome and an increasing number of metabolic comorbidities were likewise associated with progressively higher EOCRC risk, with the strongest associations observed among individuals diagnosed at younger ages [53]. However, these observational findings do not establish whether intentional weight reduction or pharmacological management of metabolic dysfunction translates into a measurable reduction in EOCRC incidence [53,57]. Nevertheless, given the consistent association between obesity and metabolic dysfunction and increased EOCRC risk, effective modification of these factors would be expected to have a favourable impact on colorectal cancer prevention. However, the magnitude of this effect on EOCRC incidence remains to be established [53,138].

Dietary and behavioural recommendations should likewise emphasise overall patterns rather than EOCRC-specific prescriptions. Current evidence supports promoting a predominantly plant-based dietary pattern rich in vegetables, whole fruits, legumes, and whole grains while limiting red and processed meat, sugar-sweetened beverages, and highly processed foods. In the systematic review by Maddula et al., Western dietary patterns characterised by higher consumption of meat, processed foods, and added sugars and lower fibre intake were consistently associated with increased EOCRC risk, with reported adjusted odds ratios ranging from 1.84 to 2.99. Sedentary behaviours were likewise associated with higher risk (aRR 1.69–2.44), whereas moderate-to-vigorous physical activity was associated with lower EOCRC risk (aOR 0.34–0.58) [57]. The American Cancer Society also recommends 150–300 min of moderate-intensity or 75–150 min of vigorous-intensity physical activity per week for adults, along with reducing prolonged sedentary behaviour and maintaining a healthy body weight [57,138]. Alcohol reduction and smoking cessation should also remain explicit targets. In a 2025 systematic review and meta-analysis including 11 studies of alcohol use and 12 studies of smoking, alcohol consumption was associated with a higher risk of EOCRC (pooled OR, 1.39; 95% CI, 1.14–1.69), with a positive dose–response relationship indicating a 2% increase in risk per additional 10 g/day of ethanol consumed (OR, 1.02; 95% CI, 1.01–1.08). Smoking was similarly associated with increased EOCRC risk (pooled OR, 1.39; 95% CI, 1.20–1.59) [139].

Collectively, these considerations support a life-course approach to EOCRC prevention. The pronounced birth-cohort effects and the occurrence of relevant metabolic and behavioural exposures early in life suggest that prevention may need to begin in childhood, adolescence, and young adulthood rather than only when individuals approach screening age [57,80,140]. Population-level strategies to prevent excess adiposity, preserve metabolic health, improve dietary quality, increase habitual physical activity, and reduce tobacco and alcohol exposure therefore represent key components of a long-term primary prevention strategy [138,139]. Nevertheless, the proportion of EOCRC that these factors could prevent remains uncertain [57,140]. Most available evidence is observational, individual risk factors often coexist, and currently identified modifiable exposures do not fully explain the observed epidemiological increase in EOCRC [57,80,140].

### 8.2. Risk-Stratified Screening and Surveillance

A central challenge in EOCRC prevention is distinguishing between risk-associated characteristics and conditions where the magnitude and clinical definition of risk justify earlier screening or dedicated surveillance [137,141]. This distinction is particularly important because EOCRC-associated exposures such as obesity and metabolic dysfunction are common even among younger adults. In contrast, the absolute incidence of colorectal cancer before age 50 remains comparatively low [142,143,144]. Current recommendations therefore support substantially different approaches for individuals with established high-risk conditions, such as a significant family history or colonic IBD, than for those with metabolic or lifestyle-related risk factors alone [137,141,145].

Family history remains one of the most clinically actionable determinants of earlier colorectal cancer screening. Individuals with a first-degree relative affected by CRC may need screening before the age recommended for the average-risk population, with the starting age and subsequent screening interval guided by factors such as the affected relative’s age at diagnosis and the number of affected family members [141,146]. Risk assessment should therefore include a detailed family history obtained before routine screening age, including the number and degree of affected relatives, their ages at diagnosis, the occurrence of multiple primary cancers, and patterns of malignancy suggestive of hereditary CRC syndromes [137,141,146]. Particularly early CRC, multiple primary cancers, clustering of colorectal cancers within a family, or a pattern of cancers suggestive of a hereditary CRC syndrome should prompt consideration of genetic counselling and germline testing rather than management solely through an earlier sporadic CRC screening schedule [137,146].

Colonic IBD is a separate, established high-risk setting in which surveillance depends primarily on disease duration and additional clinical risk factors rather than chronological age alone. Contemporary guidance recommends colonoscopic risk assessment after several years of colonic disease, with earlier surveillance in selected high-risk settings such as primary sclerosing cholangitis. Subsequent surveillance intervals are individualised based on factors including disease extent, inflammatory activity, prior dysplasia, strictures, PSC, and family history of CRC [145,147]. This should be distinguished from population screening for EOCRC: IBD surveillance is directed at preventing or detecting IBD-associated colorectal neoplasia in a defined high-risk population [145].

By contrast, obesity, metabolic syndrome, diabetes, and related metabolic abnormalities are not currently established as stand-alone indications for earlier CRC screening or colonoscopic surveillance in major clinical recommendations [137,141]. Although these characteristics are associated with EOCRC [53], their high prevalence among younger adults limits their usefulness as isolated screening criteria [143,144]. Using obesity or metabolic dysfunction alone to define eligibility for earlier screening could substantially expand the population considered for screening while including many individuals whose absolute short-term CRC risk remains low [142,143,144]. Emerging modelling studies suggest that individuals with diabetes or metabolic syndrome may reach CRC risk thresholds at younger ages, supporting further investigation of risk-adapted screening strategies [148]. However, evidence demonstrating that earlier screening based solely on metabolic characteristics improves clinical outcomes or is cost-effective remains insufficient [141,148]. Metabolic factors may therefore contribute to risk assessment but, at present, should not be considered equivalent to established high-risk conditions such as hereditary predisposition, a strong family history, or colonic IBD [137,141,145].

A potential future strategy is multivariable risk stratification, which combines several moderate-risk characteristics rather than using them individually. Risk-prediction models that incorporate family history, lifestyle and environmental factors, and polygenic risk can identify subgroups with substantially different relative risks of EOCRC. For example, combined environmental and polygenic risk scores have produced stronger risk gradients than either component alone. However, currently available models show only moderate discriminatory performance, while the absolute risk of EOCRC remains low even in many individuals classified as higher risk [149]. In addition, these tools require external validation and calibration across populations and ancestries [149,150], as well as evidence that implementing them improves clinical outcomes and is cost-effective [141].

Thus, current risk-stratified prevention should prioritise established high-risk pathways, including earlier screening for individuals with significant familial or hereditary risk and disease-specific surveillance for patients with colonic IBD [137,141,145]. Obesity and metabolic dysfunction may help refine future risk assessment but do not currently justify separate screening pathways on their own [137,141,148]. An important research objective is therefore to develop and validate integrated prediction models that can identify younger adults whose absolute CRC risk may be high enough to justify screening before the standard population threshold [141,149,150].

### 8.3. Earlier Diagnosis in Symptomatic Younger Adults

#### 8.3.1. From Symptom Recognition to Timely Diagnosis

Consider earlier detection of EOCRC separately from screening asymptomatic individuals. While population screening aims to detect premalignant lesions or asymptomatic cancers before symptoms develop, earlier diagnosis in younger adults depends primarily on timely evaluation of clinically relevant symptoms. This distinction matters because most individuals who develop CRC before the usual screening age are not identified through organised population screening but present with symptoms [137,151].

The diagnostic challenge is partly related to the relatively low absolute incidence of CRC in younger adults and the high prevalence of benign gastrointestinal and anorectal conditions [137,151]. A systematic review and meta-analysis of 81 studies involving more than 24 million individuals younger than 50 years found that hematochezia was present in approximately 45% of patients with EOCRC, abdominal pain in 40%, and altered bowel habits in 27%. Hematochezia was associated with a particularly strong increase in the likelihood of EOCRC, while anaemia and abdominal pain were also associated with increased risk. The same analysis found that the interval from symptom presentation to EOCRC diagnosis was commonly several months, with a mean of 6.4 months and a median of 4 months [151].

#### 8.3.2. Clinical Features Warranting Further Investigation

Several clinical features should prompt consideration of colorectal pathology in symptomatic younger adults. These include hematochezia or persistent rectal bleeding, particularly when recurrent or unexplained, iron-deficiency anaemia without an established alternative cause, unintentional weight loss, persistent or unexplained abdominal pain, new or persistent changes in bowel habits, including altered stool frequency or consistency, and a palpable abdominal or rectal mass. Multiple symptoms, particularly when they persist or progressively worsen, should further lower the threshold for diagnostic investigation [137,151,152,153].

Rectal bleeding warrants particular attention because benign anorectal conditions such as haemorrhoids and anal fissures are common in younger adults and may lead to premature attribution of hematochezia to a non-malignant cause. In a systematic review of EOCRC, hematochezia was the most frequently reported presenting symptom and was associated with substantially increased odds of EOCRC [151]. Therefore, identifying a plausible benign anorectal source should not automatically exclude further assessment when bleeding is persistent, recurrent, atypical, or accompanied by other alarm features. Unexplained iron-deficiency anaemia should similarly prompt evaluation for occult gastrointestinal blood loss [137,151,152,153].

Current diagnostic guidance supports using quantitative faecal immunochemical testing (FIT) as a triage tool in symptomatic adults with features such as changes in bowel habits, iron-deficiency anaemia, abdominal pain, and selected combinations of rectal bleeding, abdominal pain, and weight loss [153]. In younger adults, FIT may help identify individuals who warrant expedited investigation while limiting unnecessary invasive procedures; however, it should remain integrated with clinical assessment, and persistent or concerning symptoms should not be dismissed solely because of young age or an initially reassuring test result [137,153].

#### 8.3.3. How Diagnostic Delay May Influence Subsequent Disease Course

The principal potential consequence of delayed diagnosis is not that the delay directly accelerates tumour biology, but that it extends the period during which an existing tumour can remain undiagnosed and untreated. During this interval, a colorectal tumour may continue to enlarge locally, invade adjacent structures, involve regional lymph nodes, or develop distant metastases. Consequently, a longer interval between symptom onset and diagnosis can reduce the chance of detecting the disease at a localised stage. However, diagnostic delay alone cannot fully explain the advanced stage frequently observed in EOCRC [151,154].

This distinction is important when interpreting the relationship between diagnostic delay and prognosis. EOCRC is biologically heterogeneous, and tumour aggressiveness, molecular subtype, hereditary predisposition, tumour location, and underlying disease biology may independently influence stage and outcomes, independent of healthcare-related delay. Evidence from comparative studies nevertheless indicates that younger adults with CRC have been observed to experience diagnostic delays and have higher odds of stage III/IV disease than older patients in some datasets [154]. A systematic review found that younger adults had higher pooled odds of presenting with stage III/IV disease (OR 1.76; 95% CI, 1.52–2.03), although the evidence for differences in individual delay intervals was heterogeneous [154].

The clinically relevant objective is therefore to shorten the interval between symptom onset, first medical assessment, and definitive diagnosis, rather than to imply that earlier investigation can alter the intrinsic biological aggressiveness of an established tumour. If diagnosis occurs while the disease remains localised, patients may access potentially curative treatment before nodal or distant dissemination develops. Conversely, diagnosis after metastatic spread substantially changes treatment requirements and prognosis. However, evidence that interventions specifically designed to increase symptom awareness among younger adults reduce EOCRC mortality remains limited [151,155].

#### 8.3.4. Balancing Earlier Diagnosis with Unnecessary Investigation

Earlier diagnosis should not translate into routine colonoscopy for all asymptomatic younger adults or indiscriminate investigation of every transient gastrointestinal symptom. The absolute incidence of EOCRC remains low in the general population below routine screening ages, making population-wide diagnostic investigation inefficient and potentially exposing large numbers of young adults to unnecessary procedures [142,143,144].

The more appropriate approach is therefore symptom-triggered and risk-adapted, incorporating additional risk factors while avoiding indiscriminate invasive investigation [153]. This approach balances the need to reduce avoidable diagnostic delay against unnecessary invasive procedures while maintaining the distinction between diagnostic evaluation of symptomatic individuals and population screening of asymptomatic adults.

### 8.4. Current Screening Recommendations and Implications for EOCRC

Current recommendations show no globally uniform screening age, with differences among countries and professional societies in the recommended age of initiation and screening strategies. In the United States, the US Preventive Services Task Force recommends CRC screening for average-risk adults aged 45–75 years, with individualised decisions at ages 76–85 years. The American College of Gastroenterology likewise suggests initiation of screening at age 45 for average-risk adults [142,156]. The American Cancer Society retained age 45 in its 2026 guideline update and expanded the available screening modalities to include blood-based cell-free DNA testing. However, stool-based tests and structural examinations remain preferred because they can detect advanced precancerous lesions and contribute to CRC prevention [157].

Evidence supporting the US shift from age 50 to 45 initially relied heavily on modelling. Three CISNET microsimulation models found that most strategies identified as efficient across all three models began at age 45; compared with starting at age 50, earlier initiation generated additional life-years gained but also required more colonoscopies and produced additional procedure-related complications [158]. This trade-off is especially relevant internationally, because the value and feasibility of expanding screening to younger age groups depend on local CRC incidence, screening participation, the balance between expected benefits and harms, and available endoscopic capacity [158,159,160]. Other countries have adopted different thresholds and programme designs [156]. The fifth edition of the European Code Against Cancer recommends organised biennial FIT-based population screening at ages 50–74, with once-only endoscopy as a possible alternative within the same age range [159]. Australia has moved further toward earlier screening: its National Bowel Cancer Screening Program provides biennial FIT-based screening from age 45 to 74, with individuals aged 45–49 able to opt into the programme and those aged 50–74 routinely invited to participate [160]. These differences show that screening-age thresholds reflect population-specific epidemiology, expected benefits and harms, health-system capacity, and programme design, rather than a single biologically fixed age [158,159,160].

For EOCRC specifically, lowering average-risk screening from age 50 to 45 is important but inherently incomplete: it extends routine population screening to adults aged 45–49 but, by design, does not cover individuals who develop CRC before age 45 [141,142]. Moreover, because CRC remains uncommon at younger ages, progressively lowering a universal screening threshold would increase the number of younger adults exposed to screening, together with the associated testing burden, colonoscopy use, harms, and costs [158,160]. Taken together, current guidance and emerging evidence support a pragmatic three-component approach: maximise participation in routine screening beginning at the locally recommended age [159,160]; ensure substantially earlier screening or surveillance for established high-risk groups, including individuals with significant familial or hereditary CRC risk and patients with colonic IBD [141,145]; and continue the development and validation of risk-based pathways for adults younger than the routine screening threshold [141,149].

## 9. Research Gaps and Future Directions

Despite the growing availability of global EOCRC data, substantial heterogeneity remains across studies, reflecting differences in geographical settings, data sources, and the completeness of cancer registration [161,162]. Therefore, more harmonised, comprehensive global data are needed to improve assessments of EOCRC incidence and temporal trends worldwide. Future studies could improve the accuracy and reliability of EOCRC estimates through greater international collaboration, integration of data from multiple sources, and continued improvements in data cleaning, correction, and statistical modelling methods. Such an approach would allow more accurate estimation of differences in EOCRC incidence and temporal trends across regions and populations worldwide, while reducing the influence of differences in data quality and completeness.

Another important gap in the current evidence is the limited availability of data on early-life exposures, despite their potential relevance to EOCRC development. Although early-life adiposity and childhood dietary patterns have been associated with later colorectal cancer risk, their specific contribution to EOCRC remains insufficiently understood [86,122,163]. Further prospective studies with repeated life-course assessments of exposures are therefore needed to clarify whether the timing, duration, and cumulative burden of these exposures influence EOCRC development. Importantly, potential early-life influences may extend to the perinatal period; however, the evidence remains limited. Studies examining caesarean delivery and birth weight have not demonstrated statistically significant associations with EOCRC overall, although some findings suggest that associations may vary across subgroups [115,164]. Therefore, further research is needed to clarify whether perinatal and other early-life exposures contribute to EOCRC development and whether their effects vary across the life course.

Notably, increasing EOCRC incidence across successive birth cohorts, particularly among younger age groups, suggests that early-life exposures may contribute to the observed generational differences in risk [11]. However, changes in EOCRC incidence may reflect both cohort and period effects. Cohort effects arise when underlying risk factors are associated with particular birth cohorts. In contrast, period effects occur when changes in risk factors or other disease drivers are associated with specific calendar periods and affect individuals across age groups or birth cohorts [31]. Distinguishing between these effects may clarify whether temporal changes in EOCRC incidence relate more closely to exposures accumulated across successive generations, broader population-level changes during specific calendar periods, or a combination of both.

Another important research gap concerns the need for integrated models that consider multiple risk factors and their cumulative effects. Although most studies have examined individual risk factors separately, some recent approaches have begun to combine different risk domains. For example, an international multicentre study developed a risk prediction model that incorporates an environmental risk score based on 16 lifestyle and environmental factors, along with a polygenic risk score for EOCRC [149]. Similarly, studies of metabolic risk have shown that EOCRC risk increases with a greater number of coexisting metabolic disorders, highlighting the potential importance of cumulative burden [53,165]. Recent evidence further highlights the potential value of exposome-based approaches, with a 2026 study identifying an association between picloram exposure-related epigenetic signatures and EOCRC, illustrating how molecular markers may help capture environmental exposures that are difficult to assess directly [166]. Similarly, a recent EOCRC study integrated dietary and lifestyle factors with gut microbiota profiles. It used mediation analysis to test whether specific bacterial taxa may link lifestyle exposures to EOCRC, further illustrating the value of incorporating microbiota-related pathways into integrated risk models [127]. Future models may therefore benefit from incorporating intermediate biological pathways, including metabolic, inflammatory and microbiota-related markers, alongside multiple exposures, their timing and duration across the life course, and potential interactions between environmental, metabolic and genetic factors.

**Figure 4:**
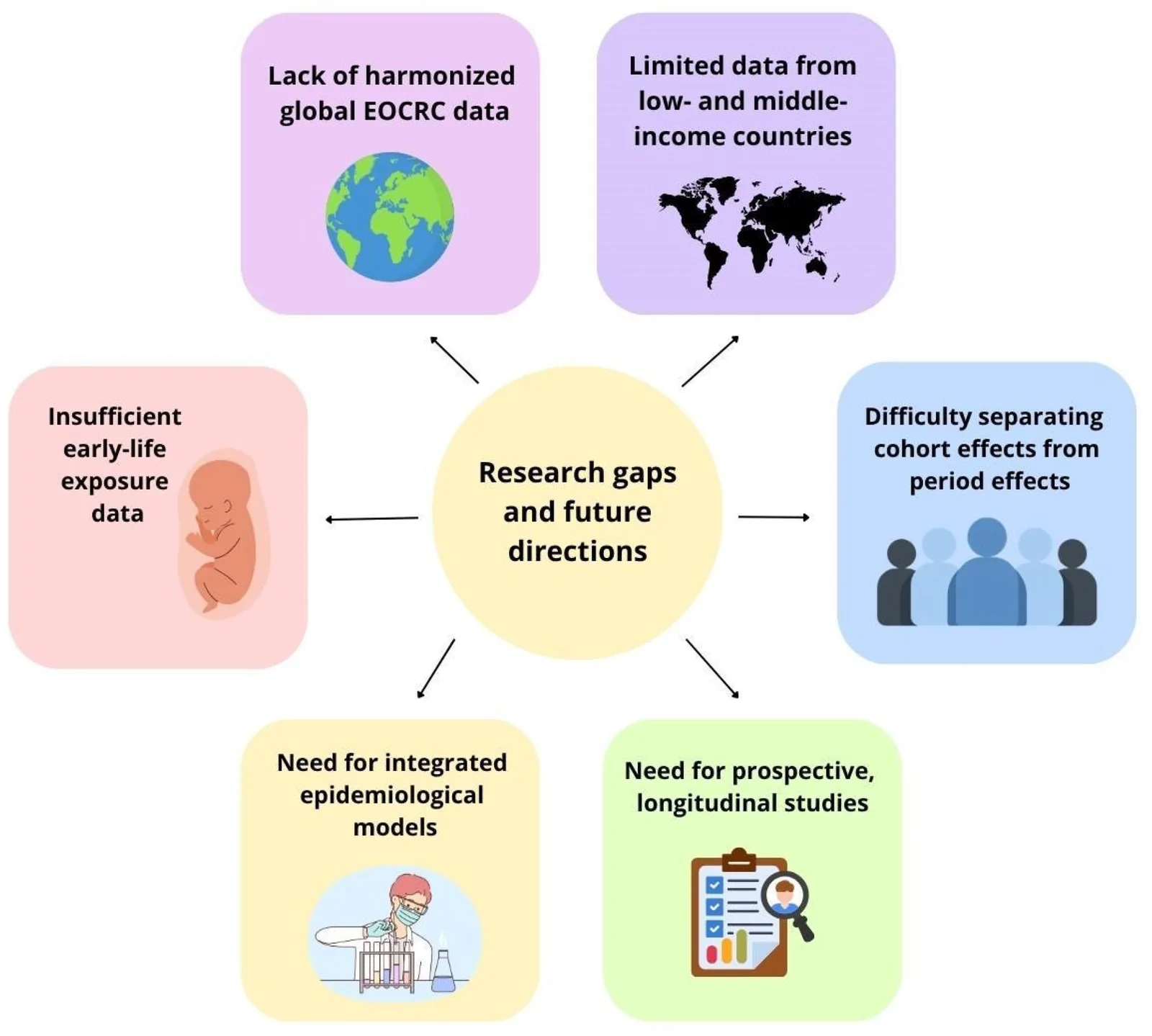
Key research gaps and future directions in EOCRC.

Finally, large-scale prospective studies are needed to characterise better the temporal relationship between risk factors and EOCRC development. Repeated measurements over the life course could help clarify the timing, duration, and cumulative burden of exposures [2,167]. However, given the relatively low incidence of EOCRC, robust analyses require sufficiently large populations and long-term follow-up to accumulate adequate numbers of cases. Although prospective designs cannot fully eliminate residual confounding, measurement error, or changes in exposure over time, they can strengthen the evidence base by providing more reliable assessment of temporal relationships and cumulative exposure patterns. Figure 4 summarises this section’s key findings and highlights the main priorities for future research.

## 10. Conclusions

Early-onset colorectal cancer is increasing in many populations but not in all. Over the most recent decade covered by the largest international comparison, incidence rose in 27 of 50 populations, remained stable in the other 23, and fell in none. The increase follows birth cohorts rather than calendar periods, and it is steepest in the youngest adults. In more than half of the populations where it occurs, incidence at ages 50 to 74 years was stable or falling at the same time. Part of the measured rise reflects changes in detection and tumour classification, including lowering the recommended screening age to 45 years in the United States and changes in the registration of neuroendocrine neoplasms. These affect the size of the trend rather than its existence. The increase persists after excluding the tumour types most sensitive to reclassification, is present in age groups no screening programme reaches, and extends to advanced-stage disease. It therefore cannot be dismissed as a measurement artefact.

Among the modifiable exposures, the strongest evidence concerns excess body weight and the metabolic dysfunction that accompanies it. Obesity has become more common in the same generations in which incidence has risen. The association also depends on when the exposure begins and how long it lasts, with raised body mass index in adolescence and in early adulthood, obesity that persists, and weight gained since early adulthood all linked to higher risk. Several other exposures act with much the same strength at every age. Whether such an exposure contributes to the widening gap between age groups therefore depends on whether it has become more common in younger generations, not on how strongly it is associated with the disease. This review reports the published attributable estimates one by one and explains why they cannot be added into a single population figure. They come from different populations, age ranges, and reference groups; the exposures overlap, and none was calculated for the intergenerational comparison the trend data describe. Therefore, the models have not quantified how much of the observed increase these exposures account for.

Projections agree in the near term and diverge beyond it. Models built differently agree that incidence will keep rising until about 2030. For 2040 and 2050 they disagree, and the disagreement comes mainly from what each model projects and how. Age-standardised rates and absolute case numbers can move in opposite directions, so a rate that levels off can still be compatible with a rising number of patients. Forecasts also differ in their baseline estimates, the age ranges they model, how they handle birth cohorts once they pass age 50, and whether they assume past trends continue. These are differences in method rather than in findings and should be read as such. The same holds geographically, because the regions with the highest rates today are not necessarily those that will produce the largest number of cases in the future.

Three points follow for practice. Lowering the routine screening age to 45 years reaches adults aged 45 to 49 but, by design, misses those who develop the disease earlier, where the steepest relative increases are seen. Below the screening age, the actionable measure is not wider use of colonoscopy in people without symptoms. It is earlier recognition of persistent symptoms, proper assessment of family history and established high-risk conditions, and prompt investigation, because most cancers arising before the screening age come to attention through symptoms. If the birth-cohort pattern holds, prevention aimed at body weight and metabolic health would have to begin well before screening age, although we do not know how much disease such measures would prevent.

The open question is no longer whether early-onset colorectal cancer is increasing, which the evidence supports, but why. The cohort pattern points to early-life exposures. The molecular and germline findings tell us what these tumours are, not why their number is rising. The population-level comparisons show that exposure and incidence have moved together, not that one causes the other. What is missing is a quantitative account that links measured changes in exposure to measured changes in incidence. Closing that gap will require harmonised international registry data, analyses that can separate cohort effects from period effects, risk models that combine exposures rather than examining them one at a time, and prospective cohorts that measure exposure repeatedly from childhood onwards. Until that evidence exists, the present literature, and therefore this review, describes the phenomenon accurately but explains it only in part.

## Figures and Tables

**Table 1:** Main EOCRC projection models and factors affecting their interpretation.

Study	Projected ASIR (Per 100,000)	95% UI for Projected ASIR	Method	Horizon	Main Result	Assumptions	Limitations	Age Range
Meng et al. [128]	6.46	5.17–7.75	Bayesian age–period–cohort (BAPC) model	2022–2030	Global ASIR projected to reach approximately 6.46 per 100,000 by 2030; incidence and prevalence increase, while mortality and DALY rates decline	Forecast based on the classic age–period–cohort framework applied to GBD 2021 data	GBD combines colon and rectal cancer; possible under-registration and underestimation in countries with limited medical and registration capacity; no data on sexual and gender minorities	20–49 years old
Zhang et al. [25]	5.42	Not reported	ARIMA model	Up to 2036	Global EOCRC incidence ASR projected to 5.42 per 100,000 in 2036, reported by the authors as a 5.56% increase from 2021; mortality ASR projected to 1.66 per 100,000	Historical time series is treated as a time-dependent stochastic process whose autocorrelation can be represented by ARIMA; the model requires stationarity	Differences in measurement standards and registration completeness across GBD settings; limited demographic/comorbidity information; age-standardisation may underestimate some age-related risks; GBD estimates depend partly on modelling and data quality	15–49 years old
Nie et al. [129]	6.70	Not reported	ARIMA model	Up to 2031	EOCRC incidence projected to reach approximately 6.70 per 100,000 by 2031	Continuation of historical time-series patterns.	Limited mechanistic interpretation and general limitations of time-series extrapolation.	<50 years old
Pan et al. [20]	5.61	Not reported	Nordpred age–period–cohort model	Up to 2040	Incidence and prevalence rates rise until a turning point in 2034 and subsequently decline; by 2040, 270,782 new EOCRC cases are projected	Extrapolation of 1990–2021 trends, assuming persistence of current age-specific incidence and mortality patterns under stable conditions	Assumes continuity of healthcare access and screening; does not incorporate future interventions, therapeutic breakthroughs, or risk-factor shifts; no uncertainty intervals	<50 years old
Li et al. [133]	Not reported	Not reported	Secondary analysis of GBD 2021 Foresight projections	2022–2050	Deaths and DALYs fluctuate but increase overall; by 2050, approximately 85,602 deaths and 4.28 million DALYs are projected	Not specified in the article; forecasts were obtained directly from the GBD 2021 Foresight tool	Primarily limited by GBD 2021 data availability and data quality; authors recommend incorporating additional data sources	15–49 years old
Tawfiles et al. [131]	Not reported	Not reported	GLOBOCAN 2022 demographic projection	2022–2050	Global absolute EOCRC incidence and mortality increase by approximately 20%; the largest increases occur in low-HDI and African regions	Constant 2022 age-standardised incidence and mortality rates applied to UN age- and sex-specific population projections.	Variable cancer-registry quality; no within-country socioeconomic stratification; does not account for future changes in healthcare access, lifestyle/environmental risk factors, or screening.	<50-year-old

## Data Availability

No new data were created or analyzed in this study. Data sharing is not applicable to this article.

## Abbreviations

The following abbreviations are used in this manuscript:

4-HNE	4-hydroxy-2-nonenal
AAPC	Average annual percentage change
ADC	Adenocarcinoma
AKT	Protein kinase B
APC	Annual percentage change
ARIMA	Autoregressive integrated moving average
ASIR	Age-standardised incidence rate
ASR	Age-standardised rate
BAPC	Bayesian age–period–cohort
BMI	Body mass index
BRICS	Brazil, Russia, India, China and South Africa
CI	Confidence interval
CIMP	CpG island methylator phenotype
CISNET	Cancer Intervention and Surveillance Modeling Network
CRC	Colorectal cancer
DALY	Disability-adjusted life year
DASH	Dietary Approaches to Stop Hypertension
EOCRC	Early-onset colorectal cancer
ERK	Extracellular signal-regulated kinase
FIT	Faecal immunochemical test
GBD	Global Burden of Disease
GLOBOCAN	Global Cancer Observatory estimates of cancer incidence and mortality
HDI	Human Development Index
HR	Hazard ratio
IBD	Inflammatory bowel disease
IGF-1	Insulin-like growth factor 1
IL-6	Interleukin 6
IRR	Incidence rate ratio
LRAU	Long-term or recurrent antibiotic use
MAPK	Mitogen-activated protein kinase
MDA	Malondialdehyde
MMP	Matrix metalloproteinase
MP	Microplastic
mTOR	Mechanistic target of rapamycin
NEN	Neuroendocrine neoplasm
NF-κB	Nuclear factor kappa B
OR	Odds ratio
PFAS	Per- and polyfluoroalkyl substances
PFOA	Perfluorooctanoic acid
PFOS	Perfluorooctane sulfonate
PI3K	Phosphoinositide 3-kinase
PKC	Protein kinase C
PRISMA	Preferred Reporting Items for Systematic Reviews and Meta-Analyses
PSC	Primary sclerosing cholangitis
RR	Relative risk
SDI	Socio-demographic Index
TMAO	Trimethylamine N-oxide
TNF-α	Tumour necrosis factor alpha
UPF	Ultra-processed food
